# The multifaceted roles of MCAM in development, homeostasis, pathological conditions, and cancer

**DOI:** 10.1007/s00109-025-02617-x

**Published:** 2025-12-12

**Authors:** Bartosz Mierzejewski, Dominika Kulma, Edyta Brzoska

**Affiliations:** Faculty of Biology, Department of Cytology, University of Warsaw, Miecznikowa 1 St, 02-096 Warsaw, Poland

**Keywords:** MCAM, Adhesion molecules, Cancer, Angiogenesis, Inflammation, Embryonic development

## Abstract

MCAM (melanoma cell adhesion molecule), identified in human melanoma in 1987, has garnered attention due to its diverse roles in development, homeostasis, and various diseases, including cancer. Initially recognized for its differential expression in tumors, MCAM plays a crucial role in cell adhesion, migration, and signaling. It acts as a receptor for multiple ligands, impacting angiogenesis, inflammation, and immune responses. MCAM is expressed in developing embryos and is implicated in trophoblast invasion during implantation, serving as a marker for placental health. In adults, MCAM is predominantly found in the vascular system and modulates endothelium homeostasis and inflammatory processes. Moreover, its involvement in cancer is marked by associations with tumor progression, particularly through epithelial-mesenchymal transition (EMT) pathways, highlighting its potential as a prognostic biomarker. Elevated levels of soluble MCAM have been linked to poor outcomes in various malignancies and can influence tumor microenvironments. This review synthesizes current understanding of MCAM’s multifunctional roles, its bidirectional influence in health and disease, and its potential as a therapeutic target in cancer.

## Introduction

MCAM (melanoma cell adhesion molecule) was discovered and described in 1987 by Johnson and co-workers in the plasma membrane of human melanoma cells [1, 2]. It was reported to be most strongly expressed in metastatic lesions and advanced primary tumors but was rarely detected in benign lesions [2]. Since then, MCAM has been described by several independent groups; it has been known under many different names such as MUC18, A32 antigen, S-Endo-1, Mel-CAM, MET-CAM, HEMCAM, or CD146 (rev. in [3, 4]). MCAM belongs to the CAM proteins—surface proteins that are involved in cell–cell and cell-extracellular matrix (ECM) adhesion. Specifically, MCAM is a part of the immunoglobulin CAM superfamily (IgSF-CAM), which are calcium-independent CAMs. Since its discovery, MCAM has been described as a crucial player in many different biological processes. As an adhesion protein, it plays a role in fish, bird, and mammal development. MCAM was also reported as a factor involved in vessel homeostasis and angiogenesis, and it was used as a pericyte and mesenchymal stromal cell/interstitial stromal cell marker. Finally, MCAM seems to be strongly involved in cancer progression, at the same time being the protein of interest in the context of antitumor therapy. In this review, we summarize the current knowledge about MCAM function, especially focusing on its role in maintaining tissue homeostasis and its role in the disease.

## MCAM in cell adhesion and signaling

The MCAM encoding gene is located on chromosome 11 in humans and on chromosome 9 in mice. MCAM was originally cloned and sequenced from a human melanoma cell cDNA library [5]. However, there is significant homology in the MCAM coding gene between mouse and human, and coding sequences are 76.2% identical [6]. So far, three forms of mouse MCAM proteins have been described: long-MCAM (MCAM-lg); short-MCAM (MCAM-sh); and soluble MCAM (s-MCAM). Two isoforms, MCAM-lg and MCAM-sh, are produced by alternative splicing of exon 15 and exhibit different intracellular domains. MCAM-lg has a long cytoplasmic tail, whereas MCAM-sh has a short cytoplasmic tail. The cytoplasmic regions of both isoforms show strong homology between humans and mice, with 93% and 95% similarity, respectively. The exon–intron structure of the human, mouse, and chicken genes is similar. The protein has a high sequence identity in human, mouse, rat, chicken, and zebrafish (rev. in [7]). s-MCAM is generated by shedding the extracellular part of MCAM-lg or MCAM-sh and can be detected in the cell culture supernatants, serum, and interstitial fluids of healthy and unhealthy subjects.

Initially, MCAM was described as an adhesion molecule by finding that melanoma cells bound to MCAM purified from melanoma cells in the solid phase [8]. Currently, it is known that the strength of MCAM adhesion is rather weak compared to other proteins such as selectins, integrins, and IgSF-CAM family members, such as ICAMs, VCAM-1, or PECAM-1 [9]. Different research groups also observed that in human cell lines, homophilic binding of MCAM is involved in the control of cell–cell adhesion [10–12]. Taira et al. report that homophilic binding of MCAM–MCAM is involved in the extension of the neurite and neuron development [13–16]. Anfosso et al. (1998, 2001) showed that MCAM phosphorylates FAK (focal adhesion kinase) through association with Fyn in endothelial cells (EC) and concluded that MCAM plays an important role in cell–cell interaction and cell migration through active actin cytoskeleton rearrangement in EC [17, 18]. Although it was initially considered to function solely as an adhesion molecule through homophilic interactions, more recent evidence indicates that MCAM also acts as a cell surface receptor for a range of ligands, including growth factors and extracellular matrix components. For example, MCAM was found to bind to several ECM-related proteins, such as laminin 411, laminin 421, galectin-1, galectin-3, or matriptase (rev. in [4]). MCAM interaction with laminin 411 facilitates lymphocyte entry into tissues and promotes inflammation [19]. On the other hand, laminin 421, but not laminin 411, was described as a putative mediator of tumor invasion and metastasis [20]. The binding of MCAM to galectin-1 protects human endothelial cells (HUVECs) from galectin-1-induced apoptosis [21]. Next, the binding of galectin-3 to MCAM results in increased migration of ECs [22]. The type II transmembrane serine protease, matriptase (MTP), was also described as an MCAM ligand. MTP-MCAM interactions have been shown to play a crucial role in the maintenance of the vascular neural cell niche. The MTP-MCAM binding results in p38/MAPK activation, GSK3β inactivation, and subsequently β-catenin activation in mouse primary brain ECs [23]. Importantly, none of these signaling events occurred when either MTP or MCAM was removed [23].

MCAM was also identified as a key player in cell signaling, with established roles in angiogenesis, vascular permeability, and leukocyte transmigration. It was found to regulate VEGFR2 as a co-receptor of VEGF [24]. The binding of VEGF to the receptor leads to the activation of many downstream signals, including FAK, which stimulates focal adhesion formation, PI3K/AKT—promoting cell survival, or MAPK and NFκB—stimulating cell proliferation [17, 25]. Similarly, MCAM was shown to act as a co-receptor of PDGDFRβ, as a crucial regulator of PDGDFRβ-dependent pericyte recruitment, and therefore, a regulator of vascular integrity maintenance [26, 27]. Also, Wnt5a uses MCAM as a receptor to regulate cell migration and convergent extension. Wnt5a binds to MCAM with the high affinity required for Wnt5a-induced activation of dishevelled kinase (Dvl) and c-jun aminoterminal kinase (JNK) in zebrafish [28]. More recent data seem to confirm results obtained using the fish model. Mouse studies suggested that, by directly binding to MCAM, Wnt5a-induced noncanonical signaling was a contributing mechanism for renal tubular inflammation in diabetic nephropathy [29]. Importantly, the direct interaction of Wnt5a and MCAM led to the activation of the JNK pathway, as was shown in HK-2 human kidney cells [29]. MCAM was also documented to act as a receptor for other ligands such as S100A8/A9, WNT1, Netrin-1, or FGF4 (extensively rev. in [4]). The ligand of MCAM which was not reviewed previously is ANGPTL2. MCAM was described as an ANGPTL2 receptor present in preadipocytes and adipocytes. MCAM ablation suppresses adipogenesis, while its loss in mature adipocytes suppresses lipid accumulation and improves energy expenditure [30]. Very recently, MCAM was also shown to regulate the stemness and chemoresistance of hepatocellular carcinoma through the activation of NFκB, resulting in an increased level of JAG2 and activation of the NOTCH pathway [31]. Collectively, MCAM acts as both an adhesive molecule and a signaling receptor that regulates many different processes mainly, but not only, related to angiogenesis and vascular function in healthy organisms and during disease.

## MCAM in vertebrate development

**Fig. 1:**
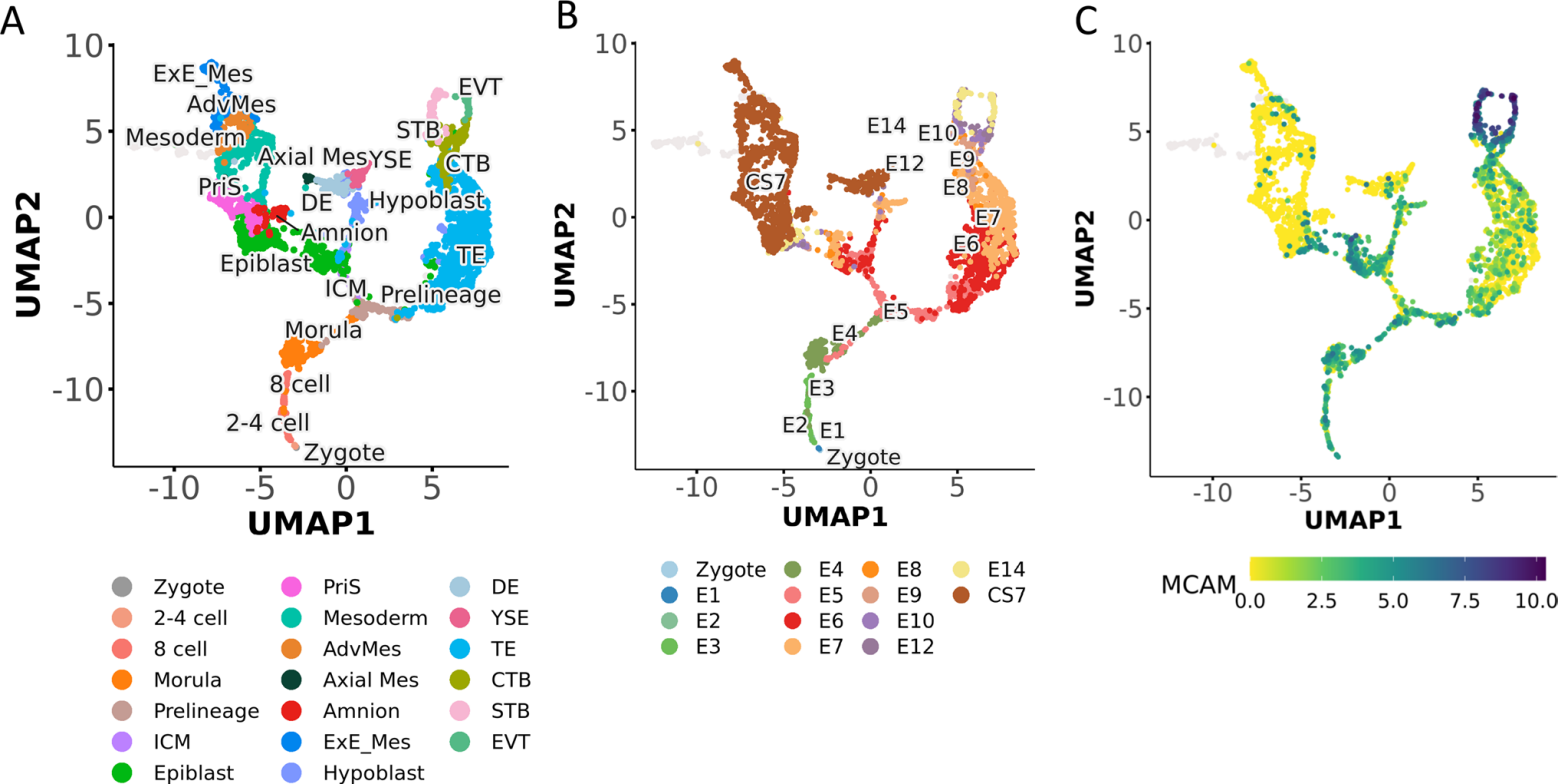
MCAM scRNA expression profile in developing and developing human embryos. **A** UMAP representing different cell types during the development of the human embryo. **B** UMAP representing the time lapse of human embryo development and **C** enrichment of MCAM expression. Visualized with a comprehensive human embryo reference tool using single-cell RNA sequencing data [32]

MCAM expression is detected in development as early as at the zygote stage and observed in the 2-cell, 8-cell, and morula stages (Fig. 1; based on a multi-data integration tool including six embryonic reference scRNA-seq data published by Zhao et al. [32]) [33–38]. Furthermore, MCAM is detected in developing embryo cell lineages, mainly in epiblast, hypoblast, and mesoderm. At the same time, it is strongly expressed in trophoblasts, specifically within syncytiotrophoblast (STB) and extravillous trophoblast (EVT) (Fig. 1). The available data suggest that MCAM plays an important role in embryo implantation and trophoblast invasion in both mice and humans. Mouse experiments showed that blocking MCAM with a function perturbation antibody AA98, before embryo implantation, caused pregnancy failure in mice [39]. In vitro studies revealed that blocking MCAM resulted in inhibition of mouse trophoblastic cell migration, decreased efficiency of mouse blastocyst attachment to the uterine luminal epithelial monolayer, decreased trophoblastic outgrowth of blastocysts, and secretion of matrix metalloproteinases [39, 40]. The importance of MCAM for trophoblast invasion was also shown in humans. Analysis of the placenta of control and pre-eclamptic patients revealed that in pre-eclampsia, intermediate trophoblasts do not express MCAM, implicating that the lack of MCAM may play a role in the development of pre-eclampsia [41]. s-MCAM has also been proposed as a biomarker in pre-eclampsia and a potential therapeutic target in a clinical trial involving more than 100 women [42]. In 2017, s-MCAM was described as a possible biomarker of embryo selection for in vitro fertilization, as embryos with the highest levels of MCAM were characterized by a significantly lower implantation rate [43]. MCAM is present not only in extraembryonic tissues but also in many embryonic tissues at different stages of development. In early human embryos (7–12 weeks of gestation), MCAM was described to be present in ECs, Schwann cells, ganglion cells, lens cells, lens smooth muscle cells, epithelial cells, glial cell fibers in the developing central nervous system, and skeletal muscles in the limbs. MCAM staining in lens epithelial cells, glial cell fibers, and skeletal muscles is not detectable in embryonic tissues after 16 weeks of gestation [44]. Importantly, MCAM was shown to be especially involved in vasculature development and function [45]. In zebrafish, in vivo knockdown of MCAM expression by morpholino severely hindered vascular development [46]. In zebrafish, mice, and humans, MCAM acts as a netrin-1 receptor, which participates in angiogenesis and morphogenesis of the vascular system. Conditional knockout of the MCAM gene in the murine or human endothelium or disruption of netrin-MCAM interaction by a specific anti-MCAM antibody blocks netrin-1-induced proliferation, migration, and in vitro or ex vivo (aortic rings assay) angiogenesis [47, 48]. In zebrafish embryos, downregulating either netrin-1a or MCAM results in very similar vascular defects [47]. More recent studies indicate that placental MCAM is dysregulated by prenatal alcohol exposure and contributes to the proangiogenic “placenta-brain” axis that controls fetal brain angiogenesis in humans and other animals [49]. In addition to its involvement in vasculature formation, MCAM was also described as a factor that regulates the development of the nervous system. In chickens, MCAM is a factor that promotes neurite extension and migration of embryonic neurons in vitro by adhesion activities [13, 14]. In mice, selective knockout of MCAM in ECs resulted in reduced levels of brain endothelial claudin-5 and blood–brain barrier (BBB) breakdown. MCAM appears to play a crucial role in controlling the behavior of endothelial cells and pericytes, thus coordinating the formation of a mature and stable BBB [27]. MCAM was also described to be crucial for the proper development of the kidney, specifically the kidney vasculature. During kidney development, MCAM-expressing cells convert to ECs expressing CD31. In embryonic kidney organ culture, inhibition of MCAM expression prevented endothelial progenitor cell proliferation and their differentiation into ECs during the development of normal vasculature [50]. Another group suggested that MCAM could participate in the increase of myocardial cell volume during the developmental growth of the rat heart [51]. Finally, very recently, MCAM was found to play an important role in the development of the mammary gland. The loss of MCAM increased the clonogenicity and regenerative capacity of mammary gland epithelial cells and promoted the proliferation, differentiation, and ductal morphogenesis of the mammary epithelium in knockout mice. *Mcam* knockout recruits and polarizes macrophages through the Il4-Stat6 axis, promoting secretion of the non-canonical Wnt ligand Wnt5a and its binding to the non-canonical Wnt signaling receptor Ryk to induce changes in mammary epithelial cells [52].

## MCAM in adult vertebrate tissues

**Fig. 2:**
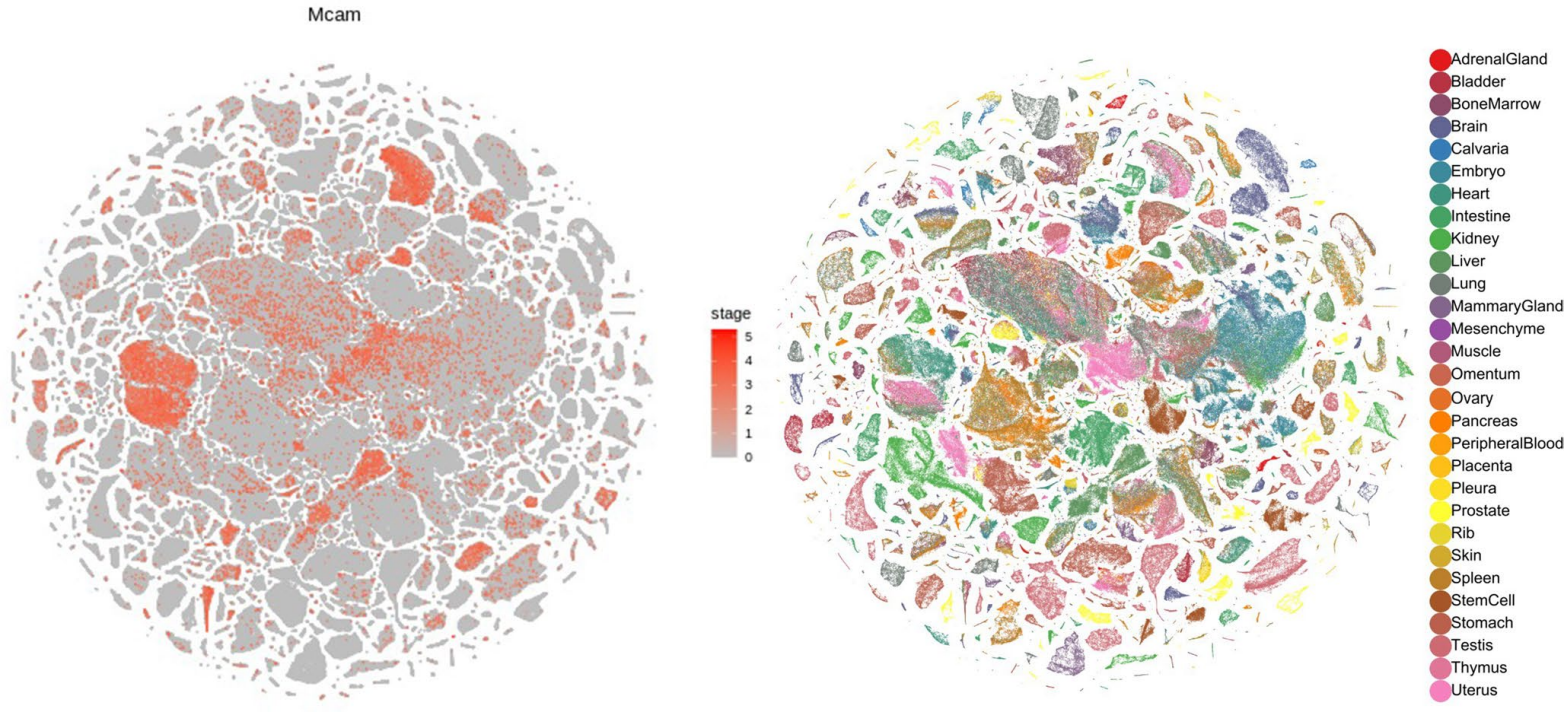
MCAM scRNA expression profile in different mouse adult cells. Colors represent cells isolated from different tissues. Generated using Mouse Cell Atlas 3.0 [55]

**Fig. 3:**
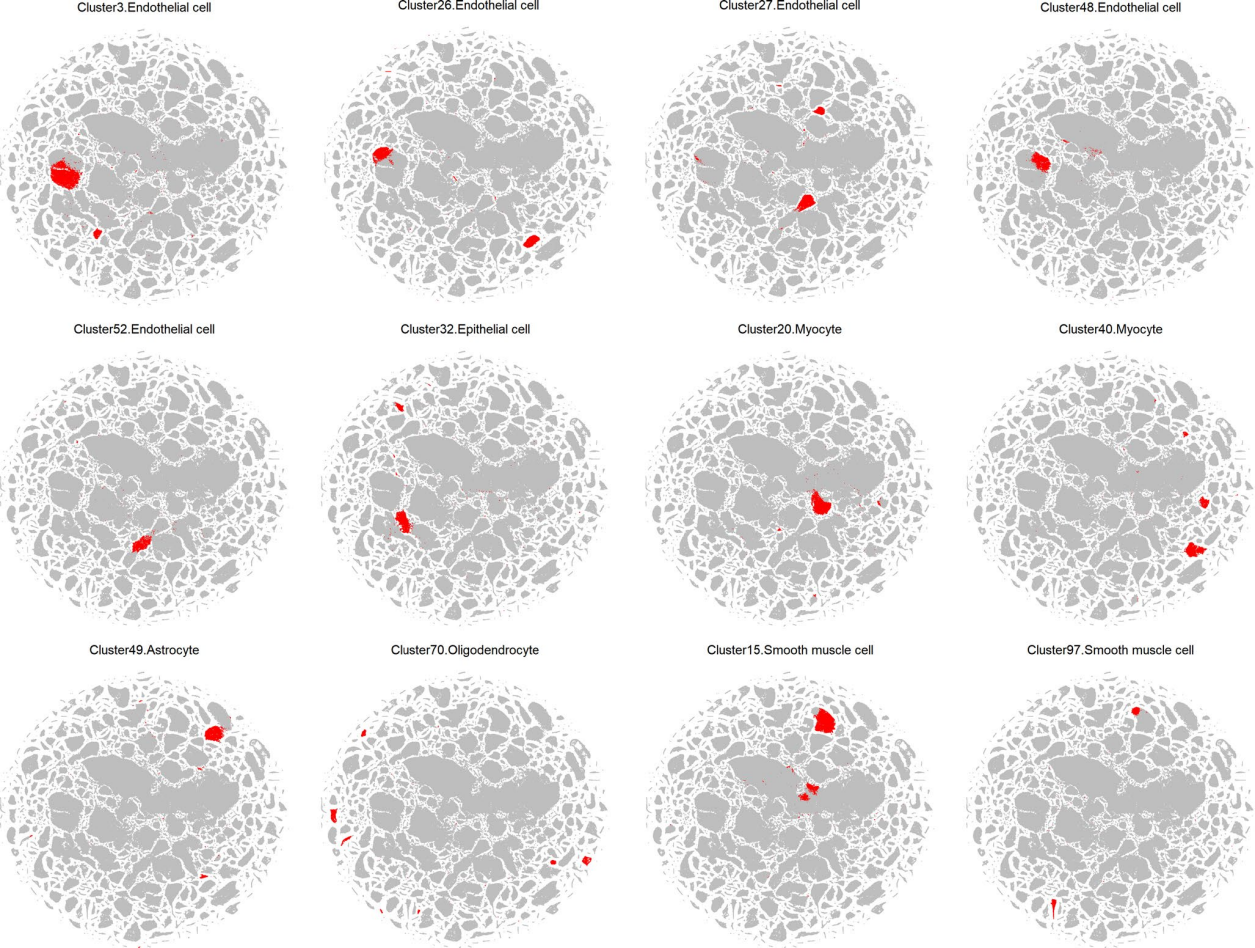
Clusters representing specific cell lineages that express MCAM in adult mouse tissues: endothelial cells, epithelial cells, myocytes, astrocytes, smooth muscle cells, and oligodendrocytes. Generated using Mouse Cell Atlas 3.0 [55]

In contrast to developmental tissues, in adult ones the expression of MCAM is rather limited. It was shown to be expressed in the endothelium, epithelium, Schwann cells, ganglion cells, cerebellar cortex, smooth muscle cells, myofibroblasts, and hair follicles [7]. However, more precise mouse single-cell RNAseq data indicates that *Mcam* expression is present in many different organs, such as the brain, kidney, liver, uterus, or skeletal muscles (Fig. 2, generated using Mouse Cell Atlas; MCA 3.0) [53–55]. The presence of *Mcam* expression was mostly detected in clusters classified as endothelial cells, epithelial cells, myocytes, astrocytes, oligodendrocytes, and smooth muscle cells (Fig. 2, Fig. 3). In adults, MCAM is involved in maintaining vessel structure, angiogenesis, and inflammation, and also serves as a marker of pericytes or the so-called mesenchymal stem cells (MSC). Importantly, MCAM expression is not stable within tissues and often is induced through external stimuli, such as inflammatory cytokines (IL-1, IL-13, TNFα), glucose or Ca2+ concentration, or growth factors (TGFβ, NGF, ET-1) (rev. in [7]).

## MCAM in the vasculature

MCAM is present in different cell types related to vasculature, ECs, smooth muscle cells (SMCs), and pericytes. An increase in MCAM synthesis was observed when HUVECs reached confluence in in vitro culture [10]. Co-labeling of MCAM with EC markers, such as PECAM1 or VE-cadherin, has shown that MCAM is present in the junctions but does not co-localize with either PECAM1 or VE-cadherin. It is located outside of tight junctions, adherent junctions, and focal adhesions [10]. Importantly, MCAM is detected not only in endothelial cells but also in the whole blood vessels, including pericytes and SMCs, and is responsible for pericyte recruitment, vessel maturation, and architecture maintenance [27]. MCAM was found to be directly upregulated by myocardin in human SMCs, together with NG2, another pericyte and SMC marker [56]. Myocardin has been previously described as a transcription factor involved in the SMC maintenance during mouse postnatal development [57]. Furthermore, MCAM was found to play a role in differentiation, proliferation, and turnover of aortic SMCs in mice [58]. Cross-regulation between MCAM and HIF-1 in SMCs triggers pulmonary vascular remodeling in mice [59]. More recent data shows that in human skin, MCAM is a specific marker of blood ECs and pericytes, but not lymphatic ECs [60]. Furthermore, the combination of MCAM+ pericytes and human dermal microvascular ECs allowed the bioengineering of a comprehensive 3D in vitro and in vivo model of the human dermal microvasculature [60]. Another study revealed that MCAM+ pericytes contribute to the formation and stabilization of the vascular network of osteogenic tissue formed in vitro in a scaffold-free construct [61].

## MCAM as a mesenchymal stem cell marker

Strictly, the term “mesenchymal stem cells” (MSC) refers to a subpopulation of MCAM+ cells in the bone marrow (BM) that was found to be able to regenerate the BM stroma and its environment after serial transplantation. When grown in vitro, BM-MSCs behave as adherent, colony-forming cells with the ability to differentiate into all skeletal tissue lineages (chondrocytes, osteoblasts) and adipocytes [62–66]. MSCs have been used in preclinical models for tissue engineering of bone, cartilage, marrow stroma, tendon, fat, and other connective tissues. Furthermore, MSCs have been shown to secrete a large spectrum of bioactive molecules, which are immunosuppressive, especially for T cells, and could be beneficial in regenerative medicine [67]. The function and potential therapeutic use of bona fide BM-derived MSCs have already been extensively reviewed many times and will not be covered by this work (e.g., [67–70]).

The high level of MCAM in MSCs has been correlated with innately higher immunomodulatory and secretory capacity and therefore therapeutic potency [71, 72]. Very quickly, similar MCAM+ perivascular cell populations, mainly pericyte-like cells, were identified in different tissues outside of BM, in multiple human and mouse organs and tissues, including skeletal muscle, pancreas, adipose tissue, and placenta. At the time, some authors suggested that pericytes might be multipotent and capable of differentiating into several cell types and are identical to MSCs present in BM [62, 73]. Recent data from human MCAM+ MSC single-cell analysis confirms their presence in different tissues and their advantages in cellular proliferation, antimicrobial activity, immune regulation, and low differentiation at the RNA level [74]. However, currently, it is also clear that both mouse and human perivascular cells present within different tissues do not behave in vivo as BM-MSCs and do not contribute to the formation of other cell types [75–78]. Although MCAM+ cells obtained from different tissues have been shown to be not the same, they may act as a local reservoir for tissue-specific progenitor cells, which may contribute to tissue reconstruction and remodeling. MCAM+ pericyte-like cells isolated from human BM, periosteum, and cord blood (CB) were able to follow the osteogenic program; however, only BM-derived cells were able to establish a hematopoietic microenvironment [77]. CB-derived MCAM+ cells were also shown to differentiate into chondrocytes [77]. Interestingly, MCAM+ pericyte-like cells obtained from human skeletal muscles could not follow the osteogenic and chondrogenic program, but differentiated very efficiently into skeletal muscle myoblasts, both in vitro and in vivo [77]*.* We and another independent group confirmed that also mouse skeletal muscle-derived MCAM+ cells possess myogenic character in vitro and can support skeletal muscle reconstruction after transplantation in vivo [79–81]. Importantly, when engrafted, these cells have a much higher capacity than NG2+ pericytes to increase Type IIa fibers recovery, capillary content, and collagen turnover after mouse hindlimb immobilization [80]. Furthermore, MCAM was also shown to not only mark specific cell subpopulations but also to have a functional impact on them.

MCAM was identified as a factor regulating cell polarity during myogenic and chondrogenic differentiation. MCAM is required in the early stages of chondrogenic differentiation and in the late stages of myogenic differentiation [82]. Also, in other tissues, MCAM has also been reported to contribute to maintaining tissue homeostasis. Similarly to skeletal muscles, MCAM was described to mark also pericyte-like cells in the human fetal and adult heart, and a small fraction of these cells was able to follow cardiomyocytic differentiation in vitro and in vivo using a mouse host [83]. MCAM+ pericyte-like cells play a crucial role in the human endometrium. The MCAM+ cell population appears to secrete growth factors (VEGF and TGF-α) and to be involved in the promotion of angiogenesis and the formation of stable blood vessel structures [84]. MCAM+ pericyte-like cells have also been described to play a crucial role in vascular regeneration after spinal cord injury, and more recently, it was shown that transplantation of human pluripotent stem cell-derived pericyte-like cells promotes functional recovery in ischemic stroke mice [85, 86].

## MCAM in inflammation

MCAM was also identified to play a role in many different inflammatory processes. First, elevated levels of MCAM are typical for active inflammatory reactions, such as idiopathic myopathy, chronically inflamed tissues, inflammatory skin disease, rheumatoid arthritis, chronic obstructive pulmonary disease, or multiple sclerosis [87–91]. A possible explanation for this phenomenon is that MCAM is mainly expressed at the endothelial junction and plays a major role in trans-endothelial migration. However, many studies have identified the contribution of MCAM to the extravasation of immune cells (rev. in [92]). First, by using MCAM inhibitory antibodies or siRNA, it was shown that s-MCAM specifically bound monocytes and HUVECs and dose-dependently increased monocyte transmigration [93]. Similarly, using anti-MCAM antibody, a significant decrease in infiltrated lymphocytes in the central nervous system (CNS) and decreased neuroinflammation in a mouse multiple sclerosis model was observed [94]. The abolition of MCAM using siRNA in pulmonary endothelial cells was associated with increased endothelial permeability and monocyte infiltration [95]. Although it is well established that the presence of vessel-associated MCAM is involved in the immune response, some studies suggest that MCAM can also be responsible for controlling the function of the immune system itself. The presence of MCAM on the surface of both inflammatory and endothelial cells may explain the differences in the results of MCAM-dependent regulation of immune cell vascular migration. In mice, MCAM is a marker of NK cell maturation, but is also detected on the surface of macrophages and a small subset of T and B cells in the periphery [96]. Furthermore, it plays an important role in the activation, differentiation, homing, and extravasation of activated immune cells. Duan et al. suggested that MCAM is an important determinant of pro-inflammatory polarization of mouse fat-derived macrophages. It was found to interact with Glycoprotein 130 (Gp130) and promote the pro-inflammatory polarization of macrophages by activating JNK signaling and inhibiting STAT3, a transcription factor crucial for anti-inflammatory polarization [97]. Another study has shown that MCAM triggers mouse macrophage activation by driving the internalization of the scavenger receptor CD36 during lipid uptake and therefore promotes foam macrophage formation [98]. Furthermore, MCAM was also described as an important regulator of macrophage migration. Blocking MCAM with a specific antibody in mice macrophages increased their migratory capacity toward the chemokines CCL19 and CCL21 [98]. The lack of MCAM in mouse macrophages resulted in impaired migration, which was mediated by reduced expression of CCR2 and suppression of the MAPK/ERK signaling pathway [99]. Except macrophages, MCAM was also detected on the surface of approximately 2–3% of the circulating T cell pool [100]. First, it was identified as an activation marker of T cells, not significantly expressed in leukocytes from healthy donors [101]. However, later, it was also described on T cells in the peripheral circulation of healthy donors. MCAM was found in both CD4+ and CD8+ T cells, as well as on a small proportion of B cells in the periphery [102]. On the functional level, MCAM+ T cells had an enhanced ability to bind the endothelial monolayers in vitro compared to MCAM cells; therefore, MCAM may improve T cell extravasation [103]. A few years later, it was demonstrated that clones of MCAM+ Th17 cells isolated from peripheral blood could adhere to endothelial cells ex vivo better than corresponding MCAM-negative Th17 cells [104]. Other studies have revealed that MCAM+ lymphocytes migrate more efficiently across the human BBB than do the corresponding MCAM- cells, and that this effect is reversed by anti-MCAM antibodies [105]. Similarly, in knockout mice lacking endothelial MCAM, the extravasation of MCAM+ T cells to the CNS was decreased compared to wild-type mice, suggesting that endothelial MCAM and lymphocytic MCAM play a role in this process [94]. Recently, it was shown that MCAM is at the center of a pathological pathway used by brain endothelial cells to recruit pathogenic CD4 + T lymphocytes from the circulation early during neuroinflammation in multiple sclerosis [106]. Furthermore, gain-of-function experiments in NK cells seem to confirm the promigratory and adhesive role of MCAM. The human NK cell line transfected with MCAM increases microvilli, decreases rolling velocity, and increases adhesion to ECs in vitro, and shows that antibodies against MCAM could reverse these effects [107].

## MCAM in disease

**Fig. 4:**
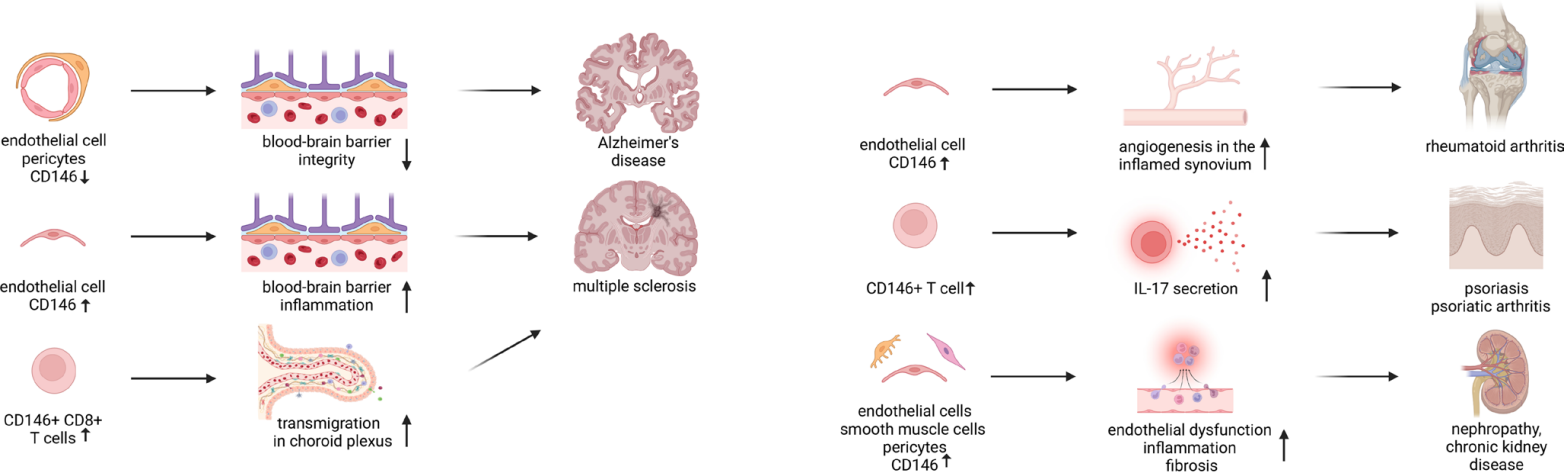
MCAM plays an important role in various pathological processes. As an adhesion molecule, MCAM helps maintain the integrity of the blood–brain barrier. Its removal during neuroinflammation is linked to increased levels of soluble MCAM, which correlate with blood–brain barrier injury and contribute to the aggressive progression of Alzheimer’s disease. In multiple sclerosis, MCAM promotes the migration of pro-inflammatory T cells across the blood–brain barrier, making it a key player in disease pathology. In rheumatoid arthritis, increased levels of soluble MCAM correlate with disease severity, while in psoriatic arthritis, MCAM+ T cells are associated with higher IL-17 production. Changes in MCAM expression also affect renal injury and are associated with various renal diseases, indicating its potential as a biomarker. Created in BioRender. Brzoska, E. (2025) https://BioRender.com/cykjnyw

As described above, MCAM plays an important role in maintaining the integrity of endothelial monolayers, but is also expressed by activated T cells, macrophages, smooth muscle cells, epithelium, fibroblasts, and MSCs. Furthermore, it plays a crucial role in inflammation and regulation of the inflammatory response. Therefore, many studies focused on its potential role in various diseases, including genetic diseases, inflammatory and auto-inflammatory diseases, and many others (Fig. 4).

MCAM is a crucial adhesion molecule for BBB integrity. The BBB is made up of pericytes, astrocytic endfeet, and endothelial cells. MCAM is located in the intercellular junctions of endothelial cells in the connections between endothelial cells and pericytes participating in the formation of the BBB [10]. Under neuroinflammatory conditions associated with failure in BBB function (e.g., injury), MCAM is not detected at the cell surface, what is accompanied with elevated levels of s-MCAM [108]. BBB dysfunction was also shown to contribute to the aggressive progression of Alzheimer’s disease. A correlation was observed between the area of the perivascular phosphorylated transactive response DNA binding protein 43 (pTDP-43) inclusions and the loss of MCAM expression. Under physiological conditions, TDP-43 is located in the cell nucleus; however, under stress conditions, it is released to the cytoplasm and hyperphosphorylated, and forms the inclusions. The accumulation of pTDP-43 in the regions of astrocytes that contact blood vessels was shown to contribute to the pathogenesis of Alzheimer's disease [109]. Most Alzheimer patients suffering from the aggressive form of the disease exhibited postmortem aggregates of pTDP-43 in their hippocampus neurons but also in astrocytes.

Furthermore, MCAM expression identifies endothelial cells with a promigratory gene signature activated during the inflammatory process in the brain, particularly in active multiple sclerosis lesions [106]. Increased expression of MCAM was shown in experimental autoimmune encephalomyelitis, a mouse model of multiple sclerosis. In vitro and in vivo studies demonstrated that MCAM in brain endothelial cells promotes the transmigration of TH1 and TH17 lymphocytes across the BBB. ST14 was identified as an immune ligand for MCAM, which was enriched in CD4+ T lymphocytes that cross the BBB in multiple sclerosis lesions. Blocking MCAM reduced the migration of ST14+ memory CD4+ T cells. Therefore, in multiple sclerosis brain ECs, MCAM plays a critical role by promoting the recruitment of pathogenic TH1 and TH17 lymphocytes, particularly ST14+ memory cells. Therefore, targeting MCAM may offer a therapeutic approach to multiple sclerosis therapy. However, MCAM expression in CD8+ T lymphocytes was shown to increase significantly during acute multiple sclerosis relapses, both in peripheral blood and cerebrospinal fluid [110]. MCAM+ CD8+ T cells exhibit an effector memory phenotype, produce pro-inflammatory cytokines, and show enhanced cytotoxic activity against myelin-producing cells, i.e., oligodendrocytes. In vitro studies showed that blocking MCAM reduces the migration of CD8+ T cells across human BBB endothelial cells. This effect was more pronounced in the presence of inflammatory cytokines. In vivo studies using mouse models of experimental autoimmune encephalomyelitis showed that MCAM inhibition or depletion reduced the severity of the disease, particularly in chronic models of multiple sclerosis. Another study demonstrated that blocking MCAM, in contrast to broad blocking of VLA-4, specifically reduced the migration of inflammatory MCAM+ T cells into the brain, particularly via the choroid plexus, which is a network of blood vessels and specialized ependymal cells located within brain ventricles and responsible for cerebrospinal fluid production [111]. This phenomenon was mediated by the interaction of MCAM with laminin 411, a major component of the endothelial basement membrane of the choroid plexus both in mouse and human. Both studies strongly implicate MCAM+ T cells as key players in the pathogenesis of multiple sclerosis.

Insoluble MCAM is also a biomarker of synovial membrane angiogenesis in rheumatoid arthritis [112]. Immunohistochemistry showed that MCAM is expressed almost exclusively in vascular ECs in synovial tissue from normal and rheumatoid arthritis patients. Significantly higher levels of s-MCAM were found in the synovial fluid of patients with rheumatoid arthritis, osteoarthritis, and psoriatic arthritis compared to healthy individuals. In patients with rheumatoid arthritis, levels of s-MCAM were significantly correlated with morning stiffness, tender joint count, and swollen joint count, but not with ESR (erythrocyte sedimentation rate) or CRP (C-reactive protein) levels. This suggests that higher levels of s-MCAM link to angiogenesis and increased EC activity rather than general inflammation in the inflamed synovium. However, a significantly higher proportion of MCAM+ CD3+ T cells was found in the synovial fluid and peripheral blood of patients with psoriatic arthritis compared to patients with rheumatoid arthritis and osteoarthritis [113]. After stimulation, MCAM+ T cells, especially memory CD4+ T cells, produced significantly higher levels of IL-17 compared to MCAM- cells. This suggests that MCAM+ T cells are a key source of IL-17 in psoriatic arthritis. Thus, MCAM plays a critical role in the pathogenesis of psoriatic arthritis by promoting the recruitment and activation of IL-17-producing T cells [114]. Furthermore, MCAM+ CD4 + T cells characterized by a high ability to secrete IL-17 were also described in peripheral blood of psoriasis patients [114]. Thus, the number of MCAM+ T cells increased in the peripheral circulation and at sites of active inflammation in patients with different autoimmune diseases such as Behcet’s disease, sarcoidosis, and inflammatory bowel disease, birdshot retinochoroidopathy [100].

As mentioned above, MCAM maintains the integrity of endothelial cell junctions. The renal endothelium is one of the most heterogeneous and highly specialized—it performs filtration, reabsorption, and transport functions. Thus, injury to this endothelium might contribute significantly to renal dysfunction. MCAM was shown to be located along the entire renal vasculature in endothelial and smooth muscle cells and pericytes, regardless of vessel size or anatomical location. Interestingly, changes in its expression were observed during renal injury, affecting various aspects of renal pathophysiology (ischemia–reperfusion, glomerulonephritis, diabetic nephropathy) [115]. Clinical studies in patients with diabetic nephropathy, IgA nephropathy, renal cell carcinoma, kidney transplants, and chronic kidney disease show correlations between MCAM expression (both tissue and soluble MCAM fractions) and disease severity, progression, and mortality. Thus, s-MCAM shows promise as a potential renal dysfunction biomarker [116]. Furthermore, MCAM upregulation was observed not only during renal disjunctions but also in primary bronchial epithelial cells of patients with chronic obstructive pulmonary disease, suggesting that it is involved in the pathophysiology of this disease [90]. These findings strongly implicate the role of MCAM in the pathogenesis of neuroinflammatory, chronic inflammatory, and arthritic kidney diseases, highlighting its potential as a therapeutic target.

## MCAM in cancer

Over the past 35 years, extensive literature that describes the expression of MCAM across various tumors has been published, including the first report identifying it in malignant melanoma cells [2, 117]. These studies range from extensively documented cases of melanoma and diverse subtypes of lung and breast cancer to more limited, less comprehensive reports on the role of MCAM in gastric cancer. The MCAM role in tumorigenesis is primarily linked to the epithelial-mesenchymal transition (EMT) and angiogenesis pathways. However, different mechanisms by which MCAM contributes to tumorigenesis are being studied. These include both the membrane-bound and soluble forms of MCAM, which may promote neoangiogenesis and metastasis. The clinical significance of MCAM is particularly notable, as it has been used as an outcome measure marker in several clinical trials focusing on kidney cancer, breast cancer, non-small cell lung cancer (NSCLC) and glioblastoma (NCT00835978, NCT00217399, NCT03493581, NCT06001281).

## Soluble MCAM

The circulatory form of MCAM, s-MCAM, level can be measured in plasma and serum. This topic has been extensively studied by researchers. In a murine xenograft model of melanoma and pancreatic cancer, s-MCAM exhibited both autocrine and paracrine pro-angiogenic and pro-tumoral effects [118]. In MCAM+ tumor cell lines, treatment with s-MCAM significantly increased cell proliferation and motility. This effect was not observed in MCAM- cells. Furthermore, treatment with s-MCAM upregulated the expression of EMT markers in two highly invasive human MCAM+ cancer cell lines—HEY (ovarian) and A375 (melanoma). These expression changes followed a similar pattern to those induced by TGF-β, a well-established EMT inducer [119]. Importantly, the same study has shown that the use of the anti-s-MCAM antibody drastically reduced metastasis but also the procoagulant activity of MCAM+ tumors. This effect was associated with a decrease in the number of circulating tumor microparticles—small vesicles released from cancer cells—and with inhibition of key signaling pathways, as shown in two in vivo models (subcutaneous xenografting and intracardiac injection of cancer cells in nude mice) [119]. Similarly, in the case of triple-negative breast cancer (TNBC), increased secretion of s-MCAM upregulated EMT markers such as SNAIL and vimentin. Blocking the soluble form with the s-MCAM-specific monoclonal antibody (M2J-1) inhibited breast tumor growth and metastasis in a xenograft mouse model [120].

## Regulatory network of MCAM expression in tumors

The expression of MCAM is subject to complex regulatory mechanisms, including epigenetic modifications and noncoding RNAs, which could influence its activity in various types of cancer. DNA methylation has been shown to suppress the expression of MCAM in certain malignancies. In breast cancer cells, treatment with the demethylating agent 5-aza-2-deoxycytidine led to increased expression of MCAM at both the mRNA and protein levels, indicating that hypermethylation of the MCAM promoter represses its transcription [121]. Aberrant CpG island methylation in the MCAM promoter has also been observed in breast cancer cell lines [121]. The MCAM gene promoter was also shown to be specifically methylated in prostate cancer cell lines and primary prostate cancer but not in non-neoplastic prostate tissues [122]. MCAM promoter methylation was directly correlated with tumor stage in primary prostate carcinoma.

On the other hand, in breast cancer, histone demethylase KDM5C—responsible for demethylating H3K4me3—has been implicated in the transcriptional control of MCAM. The TRIM11 E3 ubiquitin ligase promotes the degradation of KDM5C, thus enhancing enhancer activity at the MCAM locus and facilitating tumor cell migration and progression [123]. In rhabdomyosarcoma (RMS), the KDM3A/Ets1/MCAM axis has been identified as a key pathway, in which KDM3A promotes the expression of MCAM, contributing to tumor growth and metastasis [124]. KDM3A is a member of the Jumonji-domain histone demethylase family, and its overexpression promoted colony formation and transendothelial invasion of RMS cells. These findings underscore the potential to target histone demethylases or use demethylating agents as therapeutic strategies in cancers where MCAM plays a functional role.

Post-transcriptional regulation of MCAM is also mediated by noncoding RNAs. In melanoma, miR-516b-5p negatively regulates MCAM expression. Circular RNA circ_0079593 has been shown to act as a molecular sponge for miR-516b-5p, thus alleviating its suppression of MCAM and improving the migratory capacity of metastatic melanoma cells [125]. Furthermore, long non-coding RNA (lncRNA) uc001pwg.1 has been found to upregulate MCAM expression in endothelial cells derived from human-induced pluripotent stem cells (hiPSCs), suggesting a role in vascular development and possibly tumor angiogenesis [126].

## MCAM and cancer EMT

Multiple lines of evidence strongly suggest a link between MCAM expression and EMT. This issue is particularly relevant in the context of tumorigenesis, as EMT has been highlighted as one of the mechanisms contributing to invasion and metastasis, as described in the Hallmarks of Cancer theory [127]. EMT refers to the process by which epithelial cells acquire mesenchymal traits, such as increased migratory capacity, and could be divided into three types occurring during (1) embryonic development, (2) adult tissue regeneration, and (3) cancer progression. Although developmental EMT accompanying gastrulation and neural crest formation is tightly regulated, EMT associated with cancer progression is often characterized by dysregulation of its regulatory pathways [128].

One of the first studies aiming to specifically examine the effect of MCAM on cancer-associated EMT was that published in 2009, focusing on breast cancer cells, in which, through hierarchical cluster analysis, MCAM was defined as part of the stromal/mesenchymal gene cluster in breast cancer cell lines [129]. Moreover, MCAM knockdown correlated with reduced migration, adhesion, and proliferation of different cancer cells [130–133]. Thus, MCAM has been identified as a molecule that influences the regulation of EMT during cancer progression, and for this reason, was called the EMT inducer, specifically in breast cancer [129, 134]. The exact mechanism underlying MCAM-dependent EMT remains unclear, but numerous research papers have investigated the topic, proposing various hypotheses that often differ between specific cancer types, although some commonalities have been observed. The best-studied pathways include TGFβ and RhoA GTPase signaling, with recent studies also linking MCAM to the PI3K/AKT pathway [131, 134–136]. These signal transduction modes likely influence each other during the EMT, since RhoA is involved in TGFβ1-mediated AKT activation, as shown for breast cancer cells [137]. MCAM upregulation in cancer cells is associated with changes in EMT marker levels, such as downregulation of the epithelial marker E-cadherin and upregulation of mesenchymal markers such as vimentin, fibronectin, β-catenin, and N-cadherin [129, 131, 134, 138]. In ovarian cells, in response to TGFβ, MCAM takes part in the E-cadherin-to-N-cadherin switch, likely through the STAT3/Twist and ERK signaling pathways [131]. Overexpression of MCAM in noninvasive epithelial breast cancer cells induced migration and invasiveness associated with upregulation of EMT markers, through the RhoA pathway mediated by Slug [134]. Interestingly, quantitative proteomic analysis by Zeng et al. showed that estrogen receptor alpha (ERα) expression was significantly inhibited in luminal breast cancer cells (MCF-7), where MCAM overexpression led to increased EMT. Restoration of ERα expression in MCAM-overexpressing cells was found to negatively regulate Slug, suggesting that ERα mediates MCAM-induced EMT by suppressing Slug [139]. This finding is consistent with observations that MCAM is highly expressed in ERα-negative breast cancer cell lines [140]. A recent study has shown increased phosphorylation of PI3K and AKT in NSCLC cells overexpressing MCAM. In these cells, treatment with a PI3K inhibitor reduced the expression of mesenchymal markers and restored E-cadherin expression. This finding aligns with previous research linking MCAM expression to chemoresistance in small-cell lung cancer (SCLC), in which authors also noted its correlation with a mesenchymal phenotype, and discussed its possible involvement in EMT, considering that PI3K/AKT/SOX2 is a well-described EMT-regulating pathway [132].

## MCAM in tumor microenvironment

MCAM has also been described in the context of the tumor microenvironment. In the examined samples of malignant uveal melanoma with liver metastases, tumor cells exhibited a strong and distinct overexpression of MCAM [141]. The metastatic microenvironment was dominated by M2 macrophages, which, through their immunosuppressive and pro-angiogenic activity, supported tumor growth and progression rather than restraining it. Galectin-3 was detected predominantly in these M2 macrophages [141]. MCAM interaction with galectin-3 triggers the activation of the AKT pathway, thereby enhancing tumor cell survival, invasiveness, and the secretion of metastasis-promoting cytokines [142, 143]. Supporting this mechanism, data from uveal melanoma cell lines demonstrated colocalization of galectin-3 with MCAM on the cell surface, as well as a dose-dependent increase in AKT phosphorylation following exposure to exogenous galectin-3 [141]. MCAM has also been employed as a marker of cancer-associated fibroblasts (CAFs) [144, 145]. In a single-cell transcriptomic analysis of intrahepatic cholangiocarcinoma, an aggressive and chemoresistant malignancy, the authors identified six transcriptionally distinct fibroblast subtypes, with vascular MCAM+ cancer-associated fibroblasts (vCAFs) representing the predominant population [145]. IL-6 secreted by vCAFs induces increased expression of EZH2 (enhancer of zeste homolog 2) in intrahepatic cholangiocarcinoma cells, driving epigenetic remodeling and thereby enhancing tumor aggressiveness [145]. Moreover, studies on the lung pre-metastatic niche in liver cancer have shown that exosomes derived from hepatocellular carcinoma contain miR-1247-3p, which drives metastasis by converting fibroblasts into CAFs through the downregulation of β−1,4-galactosyltransferase, thereby activating the β1-integrin-NF-κB signaling pathway [146]. Notably, in a different study, comparative proteomic analyses of plasma-derived extracellular vesicles from melanoma patients and healthy donors identified MCAM as one of the markers elevated in patients with melanoma [147]. Recent data show that MCAM is a key cargo of EVs released by breast cancer cells and plays a crucial role in the EV-dependent seeding of pre-metastatic niches [148]. The presence of MCAM in the tumor microenvironment, within the stromal cell interactions described above, likely contributes to metastasis, consistent with its recognized role in supporting tumor aggressiveness through the promotion of malignant phenotypes.

## MCAM and tumor resistance

The issue of resistance to therapy is crucial in the treatment of patients, as the challenge of overcoming resistance remains a fundamental therapeutic challenge in both chemotherapy and personalized medicine. The study showed that MCAM expression is elevated in carboplatin-resistant SCLC xenograft tumors compared to treatment-naive ones. In chemoresistant SCLC cells, MCAM knockdown significantly reduced proliferation and suppressed activation of the PI3K/AKT pathway. Moreover, SOX2 knockdown in chemoresistant SCLC cells led to decreased MCAM expression, suggesting a regulatory link between SOX2 and MCAM [132]. Analysis of breast cancer cells revealed that MCAM is highly expressed in ERα-negative breast cancer cell lines, which are generally unresponsive to tamoxifen. In ERα-positive cells, overexpression of MCAM leads to tamoxifen resistance, whereas silencing MCAM restores tamoxifen sensitivity. MCAM-overexpressing breast cancer cells exhibit increased expression of Slug and downregulation of ERα, suggesting that the MCAM/Slug/ERα axis may contribute to tamoxifen resistance in ERα-negative breast cancer [140]. Furthermore, MCAM-positive glioblastoma cell lines showed increased proliferation, migration, and invasion after stimulation with s-MCAM [149]. Consistently, elevated levels of s-MCAM were observed in the plasma of glioblastoma patients treated with bevacizumab, i.e., anti-VEGF mAb, as well as in glioblastoma cells exposed to bevacizumab in vitro. Notably, the neutralization of s-MCAM using mucizumab, an anti-s-MCAM monoclonal antibody, restored sensitivity to bevacizumab in resistant glioblastoma models. Furthermore, the combination of these two mAbs demonstrated greater inhibitory effects in mice xenografted with glioblastoma cells compared to bevacizumab alone [149]. This led to the conclusion that s-MCAM could be a biomarker for predicting and preventing resistance to bevacizumab in MCAM-positive glioblastoma. Its potential is currently being investigated in the NCT06001281 clinical trial.

## MCAM and clinical significance

Over time, MCAM expression has been identified in various cancers, but the conclusions of most studies indicate that the presence/overexpression of MCAM is strongly associated with metastasis and a poor disease prognosis. In the context of breast cancer, an early report published in 1997 suggested a potential role for MCAM as a tumor suppressor [150]. However, more than a decade later, studies contradicted these findings by demonstrating that MCAM expression enhances motility in breast cancer cell lines and characterized it as a “prometastatic factor associated with poor prognosis histoclinical features” as described above [129]. It was shown that MCAM expression is strongly associated with high tumor grade, negative ER and PR, and TNBC in patient samples. Its expression is weaker in luminal tumors compared to basal-like tumors. Furthermore, the positive expression of MCAM is correlated with a statistically significant reduction in overall survival of the patients during the first five years (*P* = 0.0104) [129]. Furthermore, MCAM expression was identified as a potential contributor to tamoxifen resistance in ERα-negative breast cancer, as reported by Liang et al. [140]. Observations in lung cancer are consistent with breast cancer observations. For example, a study reported that the 5-year survival rate in MCAM-positive adenocarcinoma patients was 50.0%, compared to 84.4% in MCAM-negative patients [151]. Similarly, Zhang et al. found that MCAM expression was present in 46.61% of squamous cell carcinoma cases and 37.47% of adenocarcinoma cases in NSCLC and was statistically associated with a reduced 5-year survival (P = 0.037) [152]. In ovarian cancer, the expression of MCAM is associated with a higher risk of early recurrence, as indicated by a shorter time to progression (TTP) and poorer overall survival (OS). Patients with MCAM-positive tumors had a median TTP of 22 months compared to 79 months for those with MCAM-negative tumors (*P* = 0.001). Similarly, median OS was significantly reduced in MCAM-positive tumors, at 42 months, compared to 131 months in MCAM-negative tumors (*P* = 0.0003) [153]. In glioblastoma cases, patients with elevated plasma levels of s-MCAM after bevacizumab treatment exhibited poorer PFS (progression-free survival) and OS compared to nonresponding patients with lower s-MCAM levels [149]. In the case of osteosarcoma, MCAM expression was reported to be significantly higher in biopsies from patients who developed metastases within 5 years compared to those without metastases. Additionally, elevated MCAM expression was associated with osteosarcoma progression [135]. Interestingly, in contradiction, Bai et al. reported that MCAM expression was lower in clear renal cell carcinoma (cRCC) samples compared to nontumor tissues. Reduced MCAM expression was significantly associated with shorter survival times after nephrectomy [154]. Furthermore, MCAM has been shown to correlate with metastasis in both colorectal and gastric cancers and was recognized as a significant prognostic factor [138, 155]. MCAM has long been associated with metastasis in melanoma, yet studies involving larger cohorts of patients appear to be lacking [156]. However, Rapanotti et al. identified a significant correlation between MCAM-positive tumors and poor patient survival, highlighting the need for further research to confirm and expand upon these findings [157].

## Context-dependent MCAM function in cancer

As described above, discrepancies exist in MCAM’s function across cancer types [158]. A large-scale meta-analysis including 12 clinical studies with 2694 participants by Zeng et al. demonstrated a strong and significant association between high MCAM expression and poor prognosis in several malignancies, including hepatocellular carcinoma, leiomyosarcoma, esophageal squamous cell carcinoma, lung cancer, colorectal cancer, clear cell renal cell carcinoma, gastric cancer, gallbladder adenocarcinoma, breast cancer, and epithelial ovarian cancer [159]. Collectively, these data confirmed that elevated MCAM expression correlates with reduced overall survival (OS) and shorter time to progression (TTP), supporting the conclusion that high MCAM expression is generally associated with poor outcomes across solid tumors [159]. However, it should also be noted that some studies report anti-tumorigenic effects of MCAM or at least link higher levels to less severe progression. For example, in ccRCC, reduced MCAM expression was significantly associated with shorter survival times after nephrectomy [160]. Conversely, the mean MCAM expression in patients with metastatic ccRCC was significantly higher than in those with localized disease. Moreover, among patients with localized ccRCC, those who experienced recurrence displayed significantly higher MCAM expression than non-recurrent cases, and high MCAM expression correlated with a markedly higher recurrence rate [161]. Another study further identified MCAM/sMCAM as a relevant biomarker of ccRCC aggressiveness and relapse during sunitinib treatment. Similarly, contradictory data exist for breast cancer [162]. MCAM was reported to inhibit breast cancer cell–endothelial adhesion and trans endothelial migration (TEM), consistent with earlier findings suggesting a suppressive effect of MCAM on breast cancer progression. Paradoxically, increased MCAM gene expression in tumor tissue was also associated with reduced patient survival (rev. in [163]). Such contradictory observations remain incompletely understood. Several non-mutually exclusive explanations can be proposed: (1) Local microenvironmental differences. MCAM’s dual role as a cell adhesion molecule and a signaling receptor makes its functional output highly dependent on the tumor microenvironment. Mechanical stress, hypoxia, inflammatory cytokine profiles, and availability of integrin partners can profoundly alter MCAM-mediated signaling. (2) Temporal and spatial regulation. MCAM function may shift during cancer progression, acting as a tumor suppressor in early stages but promoting metastasis in advanced disease. This duality is not unique to MCAM. For example, E-cadherin is differentially expressed during ovarian cancer development: absent in ovarian surface epithelial cells, present in premalignant lesions and well-differentiated tumors, and ultimately lost in late-stage invasive disease. (3) Molecular and cellular heterogeneity of tumors. MCAM is expressed across multiple cell types and can engage diverse signaling pathways. Bulk measurements of high MCAM expression may therefore reflect small subsets of highly malignant cells expressing disproportionately high levels, rather than uniform overexpression across the tumor. Despite these complexities, the consensus from current evidence is that elevated MCAM expression is, on balance, associated with adverse prognosis [164].

## MCAM as a therapeutical target

Anti-MCAM antibodies are potent therapeutic agents with various mechanisms of action, including direct tumor inhibition, immune activation, and antiangiogenic effects. One of the major challenges in targeting MCAM is its high expression in normal vascular endothelial cells. Consequently, precise control of dosing and delivery methods is essential to minimize adverse effects such as potential damage to healthy vasculature. Anti-MCAM antibodies function through multiple mechanisms. They exert direct cytotoxic effects, block MCAM-mediated signaling, reduce angiogenesis, and recruit immune effector mechanisms. These include antibody-dependent cellular cytotoxicity (ADCC), complement-dependent cytotoxicity (CDC), and antibody-dependent cellular phagocytosis (ADCP).

Anti-MCAM antibodies can directly bind to these tumor cells and could induce apoptosis. Neuroblastoma, the most common solid extracranial tumor of childhood, originates from immature cells derived from the neural crest-derived immature cells [165]. Targeting MCAM with a polyclonal antibody has shown potent antitumor effects [165]. In both in vitro and in vivo models, anti-MCAM treatment significantly suppressed neuroblastoma cell growth and increased apoptosis. In immunodeficient mice with primary neuroblastoma xenografts, anti-MCAM antibody therapy reduced tumor progression. These findings support MCAM as a promising therapeutic target and highlight the potential of anti-MCAM antibodies as an effective intervention for high-risk neuroblastoma. Moreover, chimeric antigen receptor (CAR) therapies targeting MCAM also showed promise [166]. CAR-expressing natural killer (NK) cells targeting MCAM were characterized by significantly enhanced cytotoxic activity against MCAM+ neuroblastoma cells in vitro [166]. Moreover, anti-MCAM-CAR-NK cell injection significantly decreased tumor growth and prolonged animal survival in a neuroblastoma xenograft mouse model. Then, pairing with IL-15 agonists such as NKTR-255 improves NK cell cytotoxic activity against neuroblastoma both in vitro and in vivo*.*

The therapeutic impact of anti-MCAM CAR-NK cells can be further enhanced by combination strategies, also in the case of Ewing sarcoma (ES). MCAM is highly expressed in ES cells. In models of ES, combining anti-MCAM CAR-NK cells with NKTR-255 and CD47 blockade agents such as magrolimab significantly increased macrophage-driven tumor clearance [167]. The expression of anti-MCAM CAR significantly enhanced the NK cytotoxic effect against MCAM+ ES cells in vitro and significantly reduced lung metastasis. Importantly, NKTR-255 and magrolimab significantly improved this effect and macrophage phagocytic activity against ES cells.

As discussed above, MCAM is required for the activation of AKT, p38/MAPK, and NFκB; thus, it induces the formation of new blood vessels under pathological conditions and promotes angiogenesis during tumorigenesis [168]. This suggests that MCAM could be an effective target in anticancer therapies aimed at inhibiting angiogenesis, particularly since its inhibition or knockdown reduces the adhesion, migration, and proliferation of tumor cells [130, 169]. In 2002, ABX-MA1, the MCAM monoclonal antibody (mAb), was reported to inhibit tumorigenesis and metastasis in melanoma in the xenografted mouse model; however, in the case of osteosarcoma, it did not stop tumor growth but prevented metastasis [170, 171]. In 2003, by using another anti-MCAM mAb, AA98, inhibition and reduction of blood vessel density in xenografted mice injected with human hepatocarcinoma, leiomyosarcoma, and pancreatic cancer were achieved [169]. Furthermore, functional analyses revealed that MCAM promotes proliferation, invasion, and survival of PT cells by stabilizing the Discoidin, CUB, and LCCL domain-containing protein 2 (DCBLD2) and activating the PI3K/AKT signaling pathway [172]. Therapeutic targeting of MCAM with the mAb AA98 significantly inhibited tumor growth in both malignant organoid PT and PT patient-derived xenograft (PDX) models. Importantly, in vivo administration of an anti-MCAM human mAb reduced tumor growth and osteolytic lesion formation in bone metastasis models of prostate cancer [173]. In 2017, a monoclonal antibody called TsCD146 mAb was shown to reduce the membrane expression of MCAM on melanoma and pancreatic cancer cells by up to 25%. Furthermore, its administration significantly slowed tumor growth in mice xenografted with human melanoma and pancreatic cancer cells [174]. The soluble form of MCAM can also be targeted, as demonstrated using M2J-1 mAb, which specifically binds to s-MCAM without affecting the membrane-bound form. M2J-1 significantly reduced tumor growth and vascularization in human melanoma and pancreatic cancer xenografted in mice [118]. It was later established that the use of M2J-1 mAb could also be a promising therapeutic approach for TNBC [120].

Anti-MCAM antibodies can be combined with other agents to increase therapeutic sensitivity and combine both the anti-angiogenic and anti-tumor properties of antibodies. Cervical cancer is one of the major causes of cancer death in females worldwide. In cervical cancer, the anti- MCAM monoclonal antibody AA98 enhanced radiosensitivity, promoting apoptosis and reducing tumor cell survival [175]. Furthermore, the combination of AA98 and bevacizumab (anti-VEGF mAb) showed cumulative antitumor effects on pancreatic carcinoma growth and angiogenesis in xenografted mice [24]. Significantly inhibited angiogenesis and tumor growth and metastasis were also observed when anti-MCAM antibody AA98 was used in combination with vorinostat, which is a histone deacetylase inhibitor [176]. AA98 synergized with vorinostat could be considered a novel strategy to more effectively kill lymphoma cancer cells. Correspondingly, in colorectal cancer, especially in the angiogenic CMS4 subtype, anti-MCAM antibodies inhibited tumor growth and angiogenesis by disrupting vascular signaling [177]. This antiangiogenic effect is also relevant in uveal melanoma, where targeting MCAM downregulates VEGFR/AKT/p38/NF-κB and FAK/VE-cadherin pathways [178]. Using anti-MCAM antibody AA98 induced impaired tube formation and migration of primary human retinal microvascular endothelial cells and tube-like structure formation of uveal melanoma cells [178]. Thus, AA98 treatment markedly suppressed tumor growth and angiogenesis.

Beyond cancer, anti-MCAM antibodies have applications in other diseases. An example is systemic sclerosis (SSc), which is a chronic connective tissue disease marked by progressive fibrosis of the skin and internal organs [179]. Its pathogenesis involves vascular damage, immune dysregulation, and accumulation of fibroblast-driven extracellular matrix (ECM). In both bleomycin (BLM)-induced mouse models and SSc patients, MCAM expression is elevated in dermal fibroblasts. The genetic deletion of MCAM reduced skin fibrosis, as shown by decreased dermal thickness, collagen deposition, and myofibroblast presence. MCAM appears to promote fibrosis via the Wnt/β-catenin signaling pathway. It is required for Wnt1-induced β-catenin target gene transcription. Importantly, anti-MCAM monoclonal antibodies (AA98), which bind domains 4 to 5 of MCAM, disrupt its interaction with Wnt1, inhibiting β-catenin activation and ECM gene expression in human and murine fibroblasts [179]. On the contrary, the AA1 antibody (binding domain 1) showed no significant effects [179]. Therapeutic administration of AA98 in mice with established BLM-induced dermal fibrosis significantly reduced skin thickness, collagen content, ECM production, and fibroblast number. Furthermore, nuclear β-catenin localization and target gene expression were markedly suppressed. These findings highlight MCAM as a key mediator of fibrosis through Wnt/β-catenin signaling and suggest that its blockade with AA98 is a promising strategy to stop or reverse dermal fibrosis in SSc [179].

## Summary

MCAM has emerged as a key player in cancer biology, influencing multiple aspects of tumor progression, including epithelial-mesenchymal transition, angiogenesis, metastasis, and therapy resistance. Both its membrane-bound and soluble forms contribute to aggressive cancer phenotypes, with s-MCAM acting as a potent enhancer of tumor survival and dissemination. The strong correlation between MCAM expression and poor clinical outcomes across various tumor types underscores its potential as a prognostic marker and therapeutic target. Preclinical studies using anti-MCAM antibodies show promising results in reducing tumor growth, metastasis, and resistance to existing therapies. Further clinical validation is essential to establish MCAM-targeted therapies as viable options in precision oncology.

## Data Availability

Not applicable.

## References

[1] Lehmann JM, Holzmann B, Breitbart EW, Schmiegelow P, Riethmüller G, Johnson JP (1987) Discrimination between benign and malignant cells of melanocytic lineage by two novel antigens, a glycoprotein with a molecular weight of 113,000 and a protein with a molecular weight of 76,000. Cancer Res 47(3):841–845 3542195

[2] Lehmann JM, Riethmüller G, Johnson JP (1989) MUC18, a marker of tumor progression in human melanoma, shows sequence similarity to the neural cell adhesion molecules of the immunoglobulin superfamily. Proc Natl Acad Sci U S A 86(24):9891–9895 2602381 10.1073/pnas.86.24.9891 PMC298608

[3] Shih IM (1999) The role of CD146 (Mel-CAM) in biology and pathology. J Pathol 189(1):4–11 10451481 10.1002/(SICI)1096-9896(199909)189:1<4::AID-PATH332>3.0.CO;2-P

[4] Wang Z, Xu Q, Zhang N, Du X, Xu G, Yan X (2020) CD146, from a melanoma cell adhesion molecule to a signaling receptor. Signal Transduct Target Ther. 10.1038/s41392-020-00259-8 32782280 10.1038/s41392-020-00259-8 PMC7421905

[5] Sers C, Kirsch K, Rothbächer U, Riethmüller G, Johnson JP (1993) Genomic organization of the melanoma-associated glycoprotein MUC18: implications for the evolution of the immunoglobulin domains. Proc Natl Acad Sci U S A 90(18):8514–8518 8378324 10.1073/pnas.90.18.8514 PMC47387

[6] Yang H et al (2001) Isolation and characterization of mouse MUC18 cDNA gene, and correlation of MUC18 expression in mouse melanoma cell lines with metastatic ability. Gene 265(1):133–145. 10.1016/S0378-1119(01)00349-3 11255016 10.1016/s0378-1119(01)00349-3

[7] Wang Z, Yan X (2013) CD146, a multi-functional molecule beyond adhesion. Cancer Lett 330(2):150–162 23266426 10.1016/j.canlet.2012.11.049

[8] Shih IM, Elder DE, Speicher D, Johnson JP, Herlyn M (1994) Isolation and functional characterization of the A32 melanoma-associated antigen. Cancer Res 54(9):2514–2520 8162602

[9] Vainio O, Dunon D, Aïssi F, Dangy JP, McNagny KM, Imhof BA (1996) HEMCAM, an adhesion molecule expressed by c-kit+ hemopoietic progenitors. J Cell Biol 135(6):1655–1668 8978830 10.1083/jcb.135.6.1655 PMC2133972

[10] Bardin N et al (2001) Identification of CD146 as a component of the endothelial junction involved in the control of cell-cell cohesion. Blood 98(13):3677–3684 11739172 10.1182/blood.v98.13.3677

[11] Bardin N, Francès V, Lesaule G, Horschowski N, George F, Sampol J (1996) Identification of the S-Endo 1 endothelial-associated antigen. Biochem Biophys Res Commun 218(1):210–216 8573133 10.1006/bbrc.1996.0037

[12] Johnson JP, Bar-Eli M, Jansen B, Markhof E (1997) Melanoma progression-associated glycoprotein MUC18/MCAM mediates homotypic cell adhesion through interaction with a heterophilic ligand. Int J Cancer 73(5):769–774 9398060 10.1002/(sici)1097-0215(19971127)73:5<769::aid-ijc26>3.0.co;2-#

[13] Taira E, Takaha N, Taniura H, Kim CH, Miki N (1994) Molecular cloning and functional expression of gicerin, a novel cell adhesion molecule that binds to neurite outgrowth factor. Neuron 12(4):861–872 8161457 10.1016/0896-6273(94)90338-7

[14] Taira E, Kohama K, Tsukamoto Y, Okumura S, Miki N (2005) Gicerin/CD146 is involved in neurite extension of NGF-treated PC12 cells. J Cell Physiol 204(2):632–637 15880440 10.1002/jcp.20365

[15] Taira E, Kohama K, Tsukamoto Y, Okumura S, Miki N (2004) Characterization of Gicerin/MUC18/CD146 in the rat nervous system. J Cell Physiol 198(3):377–387 14755543 10.1002/jcp.10413

[16] Hiroi S, Tsukamoto Y, Sasaki F, Miki N, Taira E (2003) Involvement of gicerin, a cell adhesion molecule, in development and regeneration of chick sciatic nerve. FEBS Lett 554(3):311–314 14623085 10.1016/s0014-5793(03)01176-1

[17] Anfosso F et al (1998) Activation of human endothelial cells via S-endo-1 antigen (CD146) stimulates the tyrosine phosphorylation of focal adhesion kinase p125(FAK). J Biol Chem 273(41):26852–26856. 10.1074/jbc.273.41.26852 9756930 10.1074/jbc.273.41.26852

[18] Anfosso F, Bardin N, Vivier E, Sabatier F, Sampol J, Dignat-George F (2001) Outside-in signaling pathway linked to CD146 engagement in human endothelial cells. J Biol Chem 276(2):1564–1569 11036077 10.1074/jbc.M007065200

[19] Flanagan K et al (2012) Laminin-411 is a vascular ligand for MCAM and facilitates TH17 cell entry into the CNS. PLoS One 7(7):e40443 22792325 10.1371/journal.pone.0040443 PMC3391262

[20] Ishikawa T et al (2014) Laminins 411 and 421 differentially promote tumor cell migration via α6β1 integrin and MCAM (CD146). Matrix Biol 38:69–83. 10.1016/j.matbio.2014.06.002 24951930 10.1016/j.matbio.2014.06.002

[21] Jouve N et al (2013) The involvement of CD146 and its novel ligand Galectin-1 in apoptotic regulation of endothelial cells. J Biol Chem 288(4):2571–2579. 10.1074/jbc.M112.418848 23223580 10.1074/jbc.M112.418848 PMC3554924

[22] Zhang Z, Zheng Y, Wang H, Zhou Y, Tai G (2018) CD146 interacts with galectin-3 to mediate endothelial cell migration. FEBS Lett. 10.1002/1873-3468.13083 29741757 10.1002/1873-3468.13083

[23] Tung H-H, Lee S-L (2017) Physical binding of endothelial MCAM and neural transmembrane protease matriptase-novel cell adhesion in neural stem cell vascular niche. Sci Rep 7(1):4946. 10.1038/s41598-017-05131-4 28694515 10.1038/s41598-017-05131-4 PMC5504030

[24] Jiang T et al (2012) CD146 is a coreceptor for VEGFR-2 in tumor angiogenesis. Blood 120(11):2330–2339 22718841 10.1182/blood-2012-01-406108

[25] Zeng Q et al (2014) Impaired tumor angiogenesis and VEGF-induced pathway in endothelial CD146 knockout mice. Protein Cell 5(6):445–456 24756564 10.1007/s13238-014-0047-y PMC4026419

[26] Chen J et al (2018) CD146 is essential for PDGFRβ-induced pericyte recruitment. Protein Cell. 10.1007/s13238-017-0484-5 29039032 10.1007/s13238-017-0484-5 PMC6053352

[27] Chen J et al (2017) CD146 coordinates brain endothelial cell-pericyte communication for blood-brain barrier development. Proc Natl Acad Sci U S A 114(36):E7622–E7631. 10.1073/pnas.1710848114 28827364 10.1073/pnas.1710848114 PMC5594696

[28] Ye Z et al (2013) Wnt5a uses CD146 as a receptor to regulate cell motility and convergent extension. Nat Commun 4(1):2803 24335906 10.1038/ncomms3803

[29] Li X et al (2021) Wnt5a promotes renal tubular inflammation in diabetic nephropathy by binding to CD146 through noncanonical Wnt signaling. Cell Death Dis 12(1):92 33462195 10.1038/s41419-020-03377-x PMC7814016

[30] Wu Z et al (2021) CD146 is a novel ANGPTL2 receptor that promotes obesity by manipulating lipid metabolism and energy expenditure. Adv Sci 8(6):2004032. 10.1002/advs.202004032 10.1002/advs.202004032 PMC7967059 33747748

[31] Yan B et al (2025) CD146 regulates the stemness and chemoresistance of hepatocellular carcinoma via JAG2-NOTCH signaling. Cell Death Dis 16(1):150 40032820 10.1038/s41419-025-07470-x PMC11876685

[32] Zhao C et al (2025) A comprehensive human embryo reference tool using single-cell RNA-sequencing data. Nat Methods 22(1):193–206 39543283 10.1038/s41592-024-02493-2 PMC11725501

[33] Yan L et al (2013) Single-cell RNA-Seq profiling of human preimplantation embryos and embryonic stem cells. Nat Struct Mol Biol 20(9):1131–1139 23934149 10.1038/nsmb.2660

[34] Petropoulos S et al (2016) Single-cell RNA-Seq reveals lineage and X chromosome dynamics in human preimplantation embryos. Cell 165(4):1012–1026 27062923 10.1016/j.cell.2016.03.023 PMC4868821

[35] Xiang L et al (2020) A developmental landscape of 3D-cultured human pre-gastrulation embryos. Nature 577(7791):537–542 31830756 10.1038/s41586-019-1875-y

[36] Tyser RCV, Mahammadov E, Nakanoh S, Vallier L, Scialdone A, Srinivas S (2021) Single-cell transcriptomic characterization of a gastrulating human embryo. Nature 600(7888):285–289 34789876 10.1038/s41586-021-04158-y PMC7615353

[37] Yanagida A, Spindlow D, Nichols J, Dattani A, Smith A, Guo G (2021) Naive stem cell blastocyst model captures human embryo lineage segregation. Cell Stem Cell 28(6):1016-1022.e4 33957081 10.1016/j.stem.2021.04.031 PMC8189436

[38] Meistermann D et al (2021) Integrated pseudotime analysis of human pre-implantation embryo single-cell transcriptomes reveals the dynamics of lineage specification. Cell Stem Cell 28(9):1625-1640.e6 34004179 10.1016/j.stem.2021.04.027

[39] Liu Q, Zhang B, Zhao X, Zhang Y, Liu Y, Yan X (2008) Blockade of adhesion molecule CD146 causes pregnancy failure in mice. J Cell Physiol 215(3):621–626 18288634 10.1002/jcp.21341

[40] Shih I, Wang T, Wu T, Kurman RJ, Gearhart JD (1998) Expression of Mel-CAM in implantation site intermediate trophoblastic cell line, IST-1, limits its migration on uterine smooth muscle cells. J Cell Sci 111(1):2655–2664. 10.1242/jcs.111.17.2655 9701564 10.1242/jcs.111.17.2655

[41] Liu Q, Yan X, Li Y, Zhang Y, Zhao X, Shen Y (2004) Pre-eclampsia is associated with the failure of melanoma cell adhesion molecule (MCAM/CD146) expression by intermediate trophoblast. Lab Invest 84(2):221–228 14688802 10.1038/labinvest.3700033

[42] Bouvier S et al (2022) Soluble CD146 is increased in preeclampsia and interacts with galectin-1 to regulate trophoblast migration through VEGFR2 receptor. F&S Science 3(1):84–94 35559998 10.1016/j.xfss.2021.11.002

[43] Bouvier S et al (2017) Soluble CD146, an innovative and non-invasive biomarker of embryo selection for in vitro fertilization. PLoS One 12(3):e0173724 28291830 10.1371/journal.pone.0173724 PMC5349662

[44] Shih IM, Nesbit M, Herlyn M, Kurman RJ (1998) A new Mel-CAM (CD146)-specific monoclonal antibody, MN-4, on paraffin-embedded tissue. Mod Pathol 11(11):1098–1106 9831208

[45] Joshkon A et al (2020) Role of CD146 (MCAM) in physiological and pathological angiogenesis—contribution of new antibodies for therapy. Biomedicines. 10.3390/biomedicines8120633 33352759 10.3390/biomedicines8120633 PMC7767164

[46] Chan B, Sinha S, Cho D, Ramchandran R, Sukhatme VP (2005) Critical roles of CD146 in zebrafish vascular development. Dev Dyn 232(1):232–244 15580611 10.1002/dvdy.20220

[47] Tu T et al (2015) CD146 acts as a novel receptor for netrin-1 in promoting angiogenesis and vascular development. Cell Res 25(3):275–287 25656845 10.1038/cr.2015.15 PMC4349246

[48] R.-C. Jiang *et al.*, “CD146 mediates the anti-apoptotic role of Netrin-1 in endothelial progenitor cells under hypoxic conditions.,” *Mol Med Rep*, vol. 25, no. 1, Jan. 2022, 10.3892/mmr.2021.12521. 10.3892/mmr.2021.12521 PMC8600420 34738629

[49] Sautreuil C et al (2023) Expression of placental CD146 is dysregulated by prenatal alcohol exposure and contributes in cortical vasculature development and positioning of vessel-associated oligodendrocytes. Front Cell Neurosci 17:1294746 38269113 10.3389/fncel.2023.1294746 PMC10806802

[50] Halt KJ et al (2016) CD146(+) cells are essential for kidney vasculature development. Kidney Int 90(2):311–324. 10.1016/j.kint.2016.02.021 27165833 10.1016/j.kint.2016.02.021

[51] Obara M et al (2021) Expression of cell adhesion molecule, Gicerin/CD146 during the formation of heart and in the cardiac hypertrophy. Mol Cell Biochem 476(5):2021–2028 33515199 10.1007/s11010-021-04068-7

[52] Yang X et al (2024) Mcam inhibits macrophage-mediated development of mammary gland through non-canonical Wnt signaling. Nat Commun 15(1):1–15 38167296 10.1038/s41467-023-44338-0 PMC10761817

[53] Han X et al (2018) Mapping the Mouse Cell Atlas by Microwell-Seq. Cell 172(5):1091-1107.e17. 10.1016/j.cell.2018.02.001 29474909 10.1016/j.cell.2018.02.001

[54] Fei L et al (2022) Systematic identification of cell-fate regulatory programs using a single-cell atlas of mouse development. Nat Genet 54(7):1051–1061 35817981 10.1038/s41588-022-01118-8

[55] Wang R et al (2023) Construction of a cross-species cell landscape at single-cell level. Nucleic Acids Res 51(2):501–516. 10.1093/nar/gkac633 35929025 10.1093/nar/gkac633 PMC9881150

[56] Rippe C et al (2021) NG2/CSPG4, CD146/MCAM and VAP1/AOC3 are regulated by myocardin-related transcription factors in smooth muscle cells. Sci Rep 11(1):5955 33727640 10.1038/s41598-021-85335-x PMC7966398

[57] Huang J et al (2015) Myocardin is required for maintenance of vascular and visceral smooth muscle homeostasis during postnatal development. Proc Natl Acad Sci USA 112(14):4447–4452. 10.1073/pnas.1420363112 25805819 10.1073/pnas.1420363112 PMC4394251

[58] Roostalu U et al (2018) Distinct cellular mechanisms underlie smooth muscle turnover in vascular development and repair. Circ Res 122(2):267–281. 10.1161/CIRCRESAHA.117.312111 29167274 10.1161/CIRCRESAHA.117.312111 PMC5771686

[59] Luo Y et al (2019) CD146-HIF-1α hypoxic reprogramming drives vascular remodeling and pulmonary arterial hypertension. Nat Commun 10(1):3551. 10.1038/s41467-019-11500-6 31391533 10.1038/s41467-019-11500-6 PMC6686016

[60] Nanni M et al (Dec.2023) CD146 expression profile in human skin and pre-vascularized dermo-epidermal skin substitutes in vivo. J Biol Eng 17(1):1–21 36721239 10.1186/s13036-023-00327-x PMC9890844

[61] Mendes LF et al (2012) Perivascular-like cells contribute to the stability of the vascular network of osteogenic tissue formed from cell sheet-based constructs. PLoS One 7(7):e41051 22829909 10.1371/journal.pone.0041051 PMC3400580

[62] Crisan M et al (2008) A perivascular origin for mesenchymal stem cells in multiple human organs. Cell Stem Cell 3(3):301–313 18786417 10.1016/j.stem.2008.07.003

[63] Sorrentino A et al (2008) Isolation and characterization of CD146+ multipotent mesenchymal stromal cells. Exp Hematol 36(8):1035–1046. 10.1016/J.EXPHEM.2008.03.004 18504067 10.1016/j.exphem.2008.03.004

[64] Russell KC, Phinney DG, Lacey MR, Barrilleaux BL, Meyertholen KE, O’Connor KC (2010) In vitro high-capacity assay to quantify the clonal heterogeneity in trilineage potential of mesenchymal stem cells reveals a complex hierarchy of lineage commitment. Stem Cells 28(4):788–798 20127798 10.1002/stem.312

[65] Sacchetti B et al (2007) Self-renewing osteoprogenitors in bone marrow sinusoids can organize a hematopoietic microenvironment. Cell 131(2):324–336 17956733 10.1016/j.cell.2007.08.025

[66] Serafini M et al (2014) Establishment of bone marrow and hematopoietic niches in vivo by reversion of chondrocyte differentiation of human bone marrow stromal cells. Stem Cell Res 12(3):659–672. 10.1016/J.SCR.2014.01.006 24675053 10.1016/j.scr.2014.01.006

[67] Caplan AI (2007) Adult mesenchymal stem cells for tissue engineering versus regenerative medicine. J Cell Physiol 213(2):341–347. 10.1002/JCP.21200 17620285 10.1002/jcp.21200

[68] Jovic D et al (2022) A brief overview of global trends in MSC-based cell therapy. Stem Cell Rev Rep 18(5):1525–1545. 10.1007/S12015-022-10369-1 35344199 10.1007/s12015-022-10369-1 PMC8958818

[69] Pittenger MF, Discher DE, Péault BM, Phinney DG, Hare JM, Caplan AI (2019) Mesenchymal stem cell perspective: cell biology to clinical progress. NPJ Regen Med 4(1):1–15 31815001 10.1038/s41536-019-0083-6 PMC6889290

[70] Margiana R et al (2022) Clinical application of mesenchymal stem cell in regenerative medicine: a narrative review. Stem Cell Res Ther 13(1):1–22. 10.1186/S13287-022-03054-0 35902958 10.1186/s13287-022-03054-0 PMC9330677

[71] Bowles AC, Kouroupis D, Willman MA, Perucca Orfei C, Agarwal A, Correa D (2020) Signature quality attributes of CD146+ mesenchymal stem/stromal cells correlate with high therapeutic and secretory potency. Stem Cells 38(8):1034–1049 32379908 10.1002/stem.3196

[72] Ma L, Huang Z, Wu D, Kou X, Mao X, Shi S (2021) CD146 controls the quality of clinical grade mesenchymal stem cells from human dental pulp. Stem Cell Res Ther 12(1):1–16 34461987 10.1186/s13287-021-02559-4 PMC8404346

[73] Caplan AI (2008) All MSCs are pericytes? Cell Stem Cell 3(3):229–230 18786406 10.1016/j.stem.2008.08.008

[74] Huang P et al (2024) Advantages of cell proliferation and immune regulation in CD146+NESTIN+ HUMSCs: insights from single-cell RNA sequencing. Stem Cells. 10.1093/STMCLS/SXAE063 39428975 10.1093/stmcls/sxae063 PMC12199618

[75] Vezzani B, Pierantozzi E, Sorrentino V (2016) Not all pericytes are born equal: pericytes from human adult tissues present different differentiation properties. Stem Cells Dev 25(20):1549–1558 27549576 10.1089/scd.2016.0177

[76] Guimarães-Camboa N et al (2017) Pericytes of multiple organs do not behave as mesenchymal stem cells in vivo. Cell Stem Cell 20(3):345-359.e5 28111199 10.1016/j.stem.2016.12.006 PMC5337131

[77] Sacchetti B et al (2016) No identical ‘Mesenchymal Stem Cells’ at different times and sites: human committed progenitors of distinct origin and differentiation potential are incorporated as adventitial cells in microvessels. Stem Cell Reports 6(6):897–913. 10.1016/j.stemcr.2016.05.011 27304917 10.1016/j.stemcr.2016.05.011 PMC4912436

[78] Cano E, Gebala V, Gerhardt H (2017) Pericytes or mesenchymal stem cells: is that the question? Cell Stem Cell 20(3):296–297 28257708 10.1016/j.stem.2017.02.005

[79] Mierzejewski B et al (2020) Mouse CD146+ muscle interstitial progenitor cells differ from satellite cells and present myogenic potential. Stem Cell Res Ther 11(1):341 32762770 10.1186/s13287-020-01827-z PMC7409690

[80] Wu Y-F et al (2022) Optimization of a pericyte therapy to improve muscle recovery after limb immobilization. J Appl Physiol 132(4):1020–1030. 10.1152/japplphysiol.00700.2021 35175105 10.1152/japplphysiol.00700.2021 PMC8993526

[81] Munroe M et al (2019) Pericyte transplantation improves skeletal muscle recovery following hindlimb immobilization. FASEB J 33(6):7694–7706. 10.1096/fj.201802580R 31021652 10.1096/fj.201802580R PMC6529341

[82] Moreno-Fortuny A, Bragg L, Cossu G, Roostalu U (2017) MCAM contributes to the establishment of cell autonomous polarity in myogenic and chondrogenic differentiation. Biol Open 6(11):1592–1601. 10.1242/bio.027771 28923978 10.1242/bio.027771 PMC5703611

[83] Chen WCW et al (2015) Human myocardial pericytes: multipotent mesodermal precursors exhibiting cardiac specificity. Stem Cells 33(2):557–573 25336400 10.1002/stem.1868 PMC4762368

[84] Hilage P et al (2024) Characterization and angiogenic potential of CD146+ endometrial stem cells. Stem Cell Res Ther 15(1):330 39334237 10.1186/s13287-024-03918-7 PMC11438155

[85] Sun J et al (2020) Transplantation of hPSC-derived pericyte-like cells promotes functional recovery in ischemic stroke mice. Nat Commun. 10.1038/S41467-020-19042-Y 33060592 10.1038/s41467-020-19042-y PMC7566513

[86] Jin H et al (2025) Physiological insights into the role of pericytes in spinal cord injury. J Cell Physiol 240(1):e31500. 10.1002/JCP.31500 39757951 10.1002/jcp.31500 PMC11701711

[87] Figarella-Branger, D., Schleinitz, N., Boutière-Albanèse, B., Camoin, L., Bardin, N., Guis, S., Pouget, J., Cognet, C., Pellissier, J. F., & Dignat-George, F (2006) Platelet-endothelial cell adhesion molecule-1 and CD146: soluble levels and in situ expression of cellular adhesion molecules implicated in the cohesion of endothelial cells in idiopathic inflammatory myopathies. J Rheumatol, 33(8), 1623–1630 16881117

[88] Middleton J et al (2005) A comparative study of endothelial cell markers expressed in chronically inflamed human tissues: MECA-79, Duffy antigen receptor for chemokines, von Willebrand factor, CD31, CD34, CD105 and CD146. J Pathol 206(3):260–268. 10.1002/PATH.1788 15887283 10.1002/path.1788

[89] Weninger W et al (2000) Keratinocytes express the CD146 (Muc18/S-endo) antigen in tissue culture and during inflammatory skin diseases. J Invest Dermatol 115(2):219–224 10951239 10.1046/j.1523-1747.2000.00039.x

[90] Schulz C et al (2003) Upregulation of MCAM in primary bronchial epithelial cells from patients with COPD. Eur Respir J 22(3):450–456 14516134 10.1183/09031936.03.00102303

[91] Dagur PK et al (2011) MCAM-expressing CD4(+) T cells in peripheral blood secrete IL-17A and are significantly elevated in inflammatory autoimmune diseases. J Autoimmun 37(4):319–327. 10.1016/J.JAUT.2011.09.003 21959269 10.1016/j.jaut.2011.09.003 PMC3223259

[92] Leroyer AS, Blin MG, Bachelier R, Bardin N, Blot-Chabaud M, Dignat-George F (2019) CD146 (Cluster of Differentiation 146). Arterioscler Thromb Vasc Biol 39(6):1026–1033 31070478 10.1161/ATVBAHA.119.312653

[93] Bardin N et al (2009) CD146 and its soluble form regulate monocyte transendothelial migration. Arterioscler Thromb Vasc Biol 29(5):746–753. 10.1161/ATVBAHA.108.183251 19229070 10.1161/ATVBAHA.108.183251

[94] Duan H et al (2013) Targeting endothelial CD146 attenuates neuroinflammation by limiting lymphocyte extravasation to the CNS. Sci Rep 3(1):1–11 10.1038/srep01687 PMC3629416 23595028

[95] Kratzer A et al (2013) Endothelial cell adhesion molecule CD146: implications for its role in the pathogenesis of COPD. J Pathol 230(4):388–398. 10.1002/PATH.4197 23649916 10.1002/path.4197

[96] Despoix N et al (2008) Mouse CD146/MCAM is a marker of natural killer cell maturation. Eur J Immunol 38(10):2855–2864 18958886 10.1002/eji.200838469

[97] Duan H et al (2022) CD146 associates with Gp130 to control a macrophage pro‐inflammatory program that regulates the metabolic response to obesity. Adv Sci 9(13):2103719. 10.1002/ADVS.202103719 10.1002/advs.202103719 PMC9069186 35258174

[98] Luo Y et al (2017) Macrophagic CD146 promotes foam cell formation and retention during atherosclerosis. Cell Res 27(3):352–372 28084332 10.1038/cr.2017.8 PMC5339843

[99] Sun J et al (2024) CD146-dependent macrophage infiltration promotes epidural fibrosis via the Erdr1/ERK/CCR2 pathway. Int Immunopharmacol. 10.1016/J.INTIMP.2024.112528 38908086 10.1016/j.intimp.2024.112528

[100] Dagur PK, McCoy JP (May2015) Endothelial-binding, proinflammatory T cells identified by MCAM (CD146) expression: characterization and role in human autoimmune diseases. Autoimmun Rev 14(5):415 25595133 10.1016/j.autrev.2015.01.003 PMC4369459

[101] Pickl WF et al (1997) MUC18/MCAM (CD146), an activation antigen of human T lymphocytes. J Immunol 158(5):2107–2115. 10.4049/JIMMUNOL.158.5.2107 9036955

[102] Elshal MF, Khan SS, Takahashi Y, Solomon MA, McCoy JP (2005) CD146 (Mel-CAM), an adhesion marker of endothelial cells, is a novel marker of lymphocyte subset activation in normal peripheral blood. Blood 106(8):2923–2924 16204154 10.1182/blood-2005-06-2307

[103] Elshal MF et al (2007) A unique population of effector memory lymphocytes identified by CD146 having a distinct immunophenotypic and genomic profile. BMC Immunol. 10.1186/1471-2172-8-29 17999761 10.1186/1471-2172-8-29 PMC2248207

[104] Brucklacher-Waldert V, Stuerner K, Kolster M, Wolthausen J, Tolosa E (2009) Phenotypical and functional characterization of T helper 17 cells in multiple sclerosis. Brain 132(12):3329–3341 19933767 10.1093/brain/awp289

[105] Larochelle C et al (2012) Melanoma cell adhesion molecule identifies encephalitogenic T lymphocytes and promotes their recruitment to the central nervous system. Brain 135(Pt 10):2906–2924. 10.1093/BRAIN/AWS212 22975388 10.1093/brain/aws212

[106] Charabati M et al (2023) MCAM+ brain endothelial cells contribute to neuroinflammation by recruiting pathogenic CD4+ T lymphocytes. Brain 146(4):1483–1495 36319587 10.1093/brain/awac389 PMC10115172

[107] Guezguez B, Vigneron P, Lamerant N, Kieda C, Jaffredo T, Dunon D (2007) Dual role of melanoma cell adhesion molecule (MCAM)/CD146 in lymphocyte endothelium interaction: MCAM/CD146 promotes rolling via microvilli induction in lymphocyte and is an endothelial adhesion receptor. J Immunol 179(10):6673–6685 17982057 10.4049/jimmunol.179.10.6673

[108] Wang D et al (2020) Soluble CD146, a cerebrospinal fluid marker for neuroinflammation, promotes blood-brain barrier dysfunction. Theranostics 10(1):231–246 31903117 10.7150/thno.37142 PMC6929609

[109] Santiago J, Utė Dovilė P., Bank B, Wennström M (2025) Perivascular phosphorylated TDP-43 inclusions are associated with Alzheimer’s disease pathology and loss of CD146 and Aquaporin-4. Brain Pathol 35(2):e13304. 10.1111/BPA.13304 39251230 10.1111/bpa.13304 PMC11835440

[110] Larochelle C et al (2015) Melanoma cell adhesion molecule–positive CD8 T lymphocytes mediate central nervous system inflammation. Ann Neurol 78(1):39–53. 10.1002/ANA.24415 25869475 10.1002/ana.24415

[111] Breuer J et al (Aug.2018) Blockade of MCAM/CD146 impedes CNS infiltration of T cells over the choroid plexus. J Neuroinflammation 15(1):1–12 30134924 10.1186/s12974-018-1276-4 PMC6106934

[112] Neidhart M, Wehrli R, Brühlmann P, Michel BA, Gay RE, Gay S (1999) Synovial fluid CD146 (MUC18), a marker for synovial membrane angiogenesis in rheumatoid arthritis. Arthritis Rheum 42(4):622–630 10211875 10.1002/1529-0131(199904)42:4<622::AID-ANR4>3.0.CO;2-Y

[113] Raychaudhuri SK, Abria C, Raychaudhuri SP (2021) Phenotype and pathological significance of MCAM+ (CD146+) T cell subset in psoriatic arthritis. Mol Biol Rep 48(10):6787–6796. 10.1007/S11033-021-06678-2/FIGURES/5 34491483 10.1007/s11033-021-06678-2 PMC8481216

[114] Kamiyama T, Watanabe H, Iijima M, Miyazaki A, Iwamoto S (2012) Coexpression of CCR6 and CD146 (MCAM) is a marker of effector memory T-helper 17 cells. J Dermatol 39(10):838–842 22486269 10.1111/j.1346-8138.2012.01544.x

[115] Boutin L, Roger E, Gayat E, Depret F, Blot-Chabaud M, Chadjichristos CE (2024) The role of CD146 in renal disease: from experimental nephropathy to clinics. J Mol Med 102(1):11–21. 10.1007/S00109-023-02392-7/FIGURES/4 37993561 10.1007/s00109-023-02392-7

[116] Keshawarz A et al (2022) Cardiovascular disease protein biomarkers are associated with kidney function: the Framingham Heart Study. PLoS One 17(5):e0268293 35544531 10.1371/journal.pone.0268293 PMC9094507

[117] “Discrimination between benign and malignant cells of melanocytic lineage by two novel antigens, a glycoprotein with a molecular weight of 113,000 and a protein with a molecular weight of 76,0001 | Cancer Research | American Association for Cancer Research.” Accessed: May 07, 2025. [Online]. Available: https://aacrjournals.org/cancerres/article/47/3/841/492204/Discrimination-between-Benign-and-Malignant-Cells 3542195

[118] Stalin J et al (2016) Targeting soluble CD146 with a neutralizing antibody inhibits vascularization, growth and survival of CD146-positive tumors. Oncogene 35(42):5489–5500 27065325 10.1038/onc.2016.83

[119] Stalin J et al (2020) Therapeutic targeting of soluble CD146/MCAM with the M2J-1 monoclonal antibody prevents metastasis development and procoagulant activity in CD146-positive invasive tumors. Int J Cancer 147(6):1666–1679. 10.1002/ijc.32909 32022257 10.1002/ijc.32909

[120] Sharma A et al (2022) Soluble CD146 as a potential target for preventing triple negative breast cancer MDA-MB-231 cell growth and dissemination. Int J Mol Sci. 10.3390/ijms23020974 35055160 10.3390/ijms23020974 PMC8780963

[121] Dudzik P et al (2019) Aberrant promoter methylation may be responsible for the control of CD146 (MCAM) gene expression during breast cancer progression. Acta Biochim Pol 66(4):619–625 31826047 10.18388/abp.2019_2907

[122] Liu JW et al (2008) Hypermethylation of MCAM gene is associated with advanced tumor stage in prostate cancer. Prostate 68(4):418–426. 10.1002/PROS.20709 18196513 10.1002/pros.20709

[123] Xiao Q et al (2022) Regulation of KDM5C stability and enhancer reprogramming in breast cancer. Cell Death Dis 13(10):1–13 10.1038/s41419-022-05296-5 PMC9530161 36192394

[124] Sobral LM et al (2020) KDM3A/Ets1/MCAM axis promotes growth and metastatic properties in Rhabdomyosarcoma. Genes Cancer 11(1–2):53. 10.18632/GENESANDCANCER.200 32577157 10.18632/genesandcancer.200 PMC7289905

[125] De Tomi E et al (Dec.2024) New axes of interaction in Circ_0079593/miR-516b-5p network in melanoma metastasis cell lines. Genes (Basel) 15(12):1647 39766913 10.3390/genes15121647 PMC11675925

[126] Lv L, Qi H, Guo X, Ni Q, Yan Z, Zhang L (2017) Long noncoding RNA uc001pwg.1 is downregulated in neointima in arteriovenous fistulas and mediates the function of endothelial cells derived from pluripotent stem cells. Stem Cells Int 2017(1):4252974 29387090 10.1155/2017/4252974 PMC5745761

[127] Hanahan D (2022) Hallmarks of cancer: new dimensions. Cancer Discov 12(1):31–46 35022204 10.1158/2159-8290.CD-21-1059

[128] Ribatti D, Tamma R, Annese T (2020) Epithelial-mesenchymal transition in cancer: a historical overview. Transl Oncol. 10.1016/J.TRANON.2020.100773 32334405 10.1016/j.tranon.2020.100773 PMC7182759

[129] Zabouo G et al (Jan.2009) CD146 expression is associated with a poor prognosis in human breast tumors and with enhanced motility in breast cancer cell lines. Breast Cancer Res 11(1):1–14 10.1186/bcr2215 PMC2687703 19123925

[130] Kang Y, Wang F, Feng J, Yang D, Yang X, Yan X (2006) Knockdown of CD146 reduces the migration and proliferation of human endothelial cells. Cell Res 16(3):313–318. 10.1038/sj.cr.7310039 16541130 10.1038/sj.cr.7310039

[131] Ma Y et al (2018) CD146 mediates an E-cadherin-to-N-cadherin switch during TGF-β signaling-induced epithelial-mesenchymal transition. Cancer Lett 430:201–214. 10.1016/J.CANLET.2018.05.016 29777784 10.1016/j.canlet.2018.05.016

[132] Tripathi SC et al (2017) MCAM mediates chemoresistance in small-cell lung cancer via the PI3K/AKT/SOX2 signaling pathway. Cancer Res 77(16):4414–4425. 10.1158/0008-5472.CAN-16-2874 28646020 10.1158/0008-5472.CAN-16-2874 PMC5880621

[133] Zabouo G et al (2009) CD146 expression is associated with a poor prognosis in human breast tumors and with enhanced motility in breast cancer cell lines. Breast Cancer Res. 10.1186/BCR2215 19123925 10.1186/bcr2215 PMC2687703

[134] Zeng Q et al (2012) CD146, an epithelial-mesenchymal transition inducer, is associated with triple-negative breast cancer. Proc Natl Acad Sci U S A 109(4):1127–1132. 10.1073/PNAS.1111053108 22210108 10.1073/pnas.1111053108 PMC3268312

[135] Du X, Zhang Q, Wang S, Chen X, Wang Y (2022) MCAM is associated with metastasis and poor prognosis in osteosarcoma by modulating tumor cell migration. J Clin Lab Anal. 10.1002/JCLA.24214 34961985 10.1002/jcla.24214 PMC8841137

[136] Wei N et al (2024) CD146 promotes EMT-mediated migration and invasion of NSCLC via PI3K/Akt signaling pathway. Front Biosci (Landmark Ed) 29(4):140 38682195 10.31083/j.fbl2904140

[137] Bakin AV, Tomlinson AK, Bhowmick NA, Moses HL, Arteaga CL (2000) Phosphatidylinositol 3-kinase function is required for transforming growth factor beta-mediated epithelial to mesenchymal transition and cell migration. J Biol Chem 275(47):36803–36810 10969078 10.1074/jbc.M005912200

[138] Liu WF et al (2012) CD146 expression correlates with epithelial-mesenchymal transition markers and a poor prognosis in gastric cancer. Int J Mol Sci 13(5):6399–6406 22754372 10.3390/ijms13056399 PMC3382746

[139] Zeng Q et al (2014) Quantitative proteomics reveals ER-α involvement in CD146-induced epithelial-mesenchymal transition in breast cancer cells. J Proteomics 103:153–169. 10.1016/J.JPROT.2014.03.033 24704855 10.1016/j.jprot.2014.03.033

[140] Liang YK et al (2017) MCAM/CD146 promotes tamoxifen resistance in breast cancer cells through induction of epithelial-mesenchymal transition, decreased ERα expression and AKT activation. Cancer Lett 386:65–76. 10.1016/J.CANLET.2016.11.004 27838413 10.1016/j.canlet.2016.11.004

[141] Krishna Y et al (2020) Transcriptome profiling reveals new insights into the immune microenvironment and upregulation of novel biomarkers in metastatic uveal melanoma. Cancers 12(10):2832 33008022 10.3390/cancers12102832 PMC7650807

[142] Pang Y et al (2022) Galectin-3 is a natural binding ligand of MCAM (CD146, MUC18) in melanoma cells and their interaction promotes melanoma progression. Biomolecules 12(10):1451. 10.3390/BIOM12101451 36291660 10.3390/biom12101451 PMC9599063

[143] Colomb F et al (2017) Galectin-3 interacts with the cell-surface glycoprotein CD146 (MCAM, MUC18) and induces secretion of metastasis-promoting cytokines from vascular endothelial cells. J Biol Chem 292(20):8381–8389. 10.1074/JBC.M117.783431 28364041 10.1074/jbc.M117.783431 PMC5437243

[144] Von Ahrens D, Bhagat TD, Nagrath D, Maitra A, Verma A (2017) The role of stromal cancer-associated fibroblasts in pancreatic cancer. J Hematol Oncol. 10.1186/S13045-017-0448-5 28351381 10.1186/s13045-017-0448-5 PMC5371211

[145] Zhang M et al (2020) Single-cell transcriptomic architecture and intercellular crosstalk of human intrahepatic cholangiocarcinoma. J Hepatol 73(5):1118–1130. 10.1016/j.jhep.2020.05.039 32505533 10.1016/j.jhep.2020.05.039

[146] Fang T et al (2018) Tumor-derived exosomal miR-1247-3p induces cancer-associated fibroblast activation to foster lung metastasis of liver cancer. Nat Commun. 10.1038/s41467-017-02583-0 29335551 10.1038/s41467-017-02583-0 PMC5768693

[147] Lattmann E et al (2024) Size-exclusion chromatography combined with DIA-MS enables deep proteome profiling of extracellular vesicles from melanoma plasma and serum. Cell Mol Life Sci 81(1):1–20. 10.1007/S00018-024-05137-Y 10.1007/s00018-024-05137-y PMC10867102 38353833

[148] Ghoroghi S et al (Jan.2021) Ral GTPases promote breast cancer metastasis by controlling biogenesis and organ targeting of exosomes. Elife 10:1–29 10.7554/eLife.61539 PMC7822591 33404012

[149] Joshkon A et al (2022) Soluble CD146, a biomarker and a target for preventing resistance to anti-angiogenic therapy in glioblastoma. Acta Neuropathol Commun 10(1):1–12 (**FIGURES/6.**) 36274147 10.1186/s40478-022-01451-3 PMC9590138

[150] I. M. Shih, M. Y. Hsu, J. P. Palazzo, and M. Herlyn (Sep. 1997) “The cell-cell adhesion receptor Mel-CAM acts as a tumor suppressor in breast carcinoma,” Am J Pathol vol. 151(no. 3): p. 745. Accessed: May 07, 2025. [Online]. Available: https://pmc.ncbi.nlm.nih.gov/articles/PMC1857834/PMC1857834 9284823

[151] Oka S, Uramoto H, Chikaishi Y, Tanaka F (2012) The expression of CD146 predicts a poor overall survival in patients with adenocarcinoma of the lung. Anticancer Res 32(3):861 22399604

[152] Zhang X, Wang Z, Kang Y, Li X, Ma X, Ma L (2014) MCAM expression is associated with poor prognosis in non-small cell lung cancer. Clin Transl Oncol 16(2):178–183 23749325 10.1007/s12094-013-1057-6

[153] Aldovini D et al (2006) M-CAM expression as marker of poor prognosis in epithelial ovarian cancer. Int J Cancer 119(8):1920–1926. 10.1002/IJC.22082 16804906 10.1002/ijc.22082

[154] Q. Bai et al (2015) “Decreased expression of mucin 18 is associated with unfavorable postoperative prognosis in patients with clear cell renal cell carcinoma,”. Int J Clin Exp Pathol vol. 8, no. 9, p. 11005. Accessed: May 07, 2025. [Online]. Available: https://pmc.ncbi.nlm.nih.gov/articles/PMC4637633/PMC4637633 26617818

[155] Tian B, Zhang Y, Li N (2013) CD146 protein as a marker to predict postoperative liver metastasis in colorectal cancer. Cancer Biother Radiopharm 28(6):466–470 23745687 10.1089/cbr.2012.1426

[156] Luca M, Hunt B, Bucana CD, Johnson JP, Fidler IJ, Bar-Eli M (1993) Direct correlation between MUC18 expression and metastatic potential of human melanoma cells. Melanoma Res 3(1):35–41 8471835 10.1097/00008390-199304000-00006

[157] Rapanotti MC et al (2014) Sequential molecular analysis of circulating MCAM/MUC18 expression: a promising disease biomarker related to clinical outcome in melanoma. Arch Dermatol Res 306(6):527–537 24902661 10.1007/s00403-014-1473-7 PMC4107285

[158] Ouhtit A et al (2009) Towards understanding the mode of action of the multifaceted cell adhesion receptor CD146. Biochim Biophys Acta 1795(2):130–136. 10.1016/J.BBCAN.2009.01.002 19356677 10.1016/j.bbcan.2009.01.002

[159] Zeng P et al (2017) Prognostic value of CD146 in solid tumor: a systematic review and meta-analysis. Sci Rep 7(1):1–7 28652617 10.1038/s41598-017-01061-3 PMC5484668

[160] Q Bai et al (2015) “Decreased expression of mucin 18 is associated with unfavorable postoperative prognosis in patients with clear cell renal cell carcinoma,”. Int J Clin Exp Pathol vol. 8(no. 9):p. 11005. Accessed: Sep. 26, 2025. [Online]. Available: https://pmc.ncbi.nlm.nih.gov/articles/PMC4637633/PMC4637633 26617818

[161] Feng G, Fang F, Liu C, Zhang F, Huang H, Pu C (2012) CD146 gene expression in clear cell renal cell carcinoma: a potential marker for prediction of early recurrence after nephrectomy. Int Urol Nephrol 44(6):1663–1669 22826148 10.1007/s11255-012-0255-4

[162] Dufies M et al (2018) Soluble CD146 is a predictive marker of pejorative evolution and of sunitinib efficacy in clear cell renal cell carcinoma. Theranostics 8(9):2447–2458 29721091 10.7150/thno.23002 PMC5928901

[163] Mannion AJ, Odell AF, Baker SM, Matthews LC, Jones PF, Cook GP (2023) Pro- and anti-tumour activities of CD146/MCAM in breast cancer result from its heterogeneous expression and association with epithelial to mesenchymal transition. Front Cell Dev Biol 11:1129015 37138793 10.3389/fcell.2023.1129015 PMC10150653

[164] Wu GJ (2012) Dual roles of METCAM in the progression of different cancers. J Oncol 2012:853797 22545053 10.1155/2012/853797 PMC3321465

[165] Obu S et al (2021) CD146 is a potential immunotarget for neuroblastoma. Cancer Sci 112(11):4617–4626. 10.1111/CAS.15124 34464480 10.1111/cas.15124 PMC8586675

[166] Luo W et al (2024) Combinatorial immunotherapy of anti-MCAM CAR-modified expanded natural killer cells and NKTR-255 against neuroblastoma. Molecular Therapy Oncology 32(4):200894. 10.1016/j.omton.2024.200894 39554906 10.1016/j.omton.2024.200894 PMC11567912

[167] Luo W et al (2024) Circumventing resistance within the Ewing sarcoma microenvironment by combinatorial innate immunotherapy. J Immunother Cancer. 10.1136/JITC-2024-009726 39266215 10.1136/jitc-2024-009726 PMC11404285

[168] Zheng C et al (2009) Endothelial CD146 is required for in vitro tumor-induced angiogenesis: the role of a disulfide bond in signaling and dimerization. Int J Biochem Cell Biol 41(11):2163–2172. 10.1016/J.BIOCEL.2009.03.014 19782948 10.1016/j.biocel.2009.03.014

[169] Yan X (2003) A novel anti-CD146 monoclonal antibody, AA98, inhibits angiogenesis and tumor growth. Blood 102(1):184–191 12609848 10.1182/blood-2002-04-1004

[170] McGary EC, Chelouche Lev D, Bar-Eli M (2002) Cellular adhesion pathways and metastatic potential of human melanoma. Cancer Biol Ther 1(5):459–465 12496470 10.4161/cbt.1.5.158

[171] McGary EC, Heimberger A, Mills L, Weber K, Thomas GW, Shtivelband M, Lev DC, Bar-Eli M (2003) A fully human antimelanoma cellular adhesion molecule/MUC18 antibody inhibits spontaneous pulmonary metastasis of osteosarcoma cells *in vivo*. Clinical cancer research : an official journal of the American Association for Cancer Research 9(17):6560–6566 14695161

[172] J Chen et al (Nov. 2023) CD146 promotes malignant progression of breast phyllodes tumor through suppressing DCBLD2 degradation and activating the AKT pathway, Cancer Commun (Lond) vol. 43(no. 11): pp. 1244–1266. 10.1002/CAC2.12495 10.1002/cac2.12495 PMC10631482 37856423

[173] E Zoni et al (May 2019) Therapeutic targeting of CD146/MCAM reduces bone metastasis in prostate cancer. Mol Cancer Res vol. 17(no. 5):pp. 1049–1062. 10.1158/1541-7786.MCR-18-1220/81519/AM/THERAPEUTIC-TARGETING-OF-CD146-MCAM-REDUCES-BONE 30745464 10.1158/1541-7786.MCR-18-1220

[174] M Nollet et al (2017) A novel anti-CD146 antibody specifically targets cancer cells by internalizing the molecule. Oncotarget vol. 8(no. 68):p. 112283. 10.18632/ONCOTARGET.22736 29348825 10.18632/oncotarget.22736 PMC5762510

[175] H Cheng (Sep. 2016) Inhibiting CD146 by its Monoclonal Antibody AA98 Improves Radiosensitivity of Cervical Cancer Cells. Med Sci Monit (vol. 22):pp. 3328–3333. 10.12659/MSM.896731 27647179 10.12659/MSM.896731 PMC5032850

[176] X Ma et al (Nov. 2010) Synergistic killing effect between vorinostat and target of CD146 in malignant cells, Clin Cancer Res vol. 16(no. 21):pp. 5165–5176. 10.1158/1078-0432.CCR-09-3174 20884621 10.1158/1078-0432.CCR-09-3174

[177] J Stalin et al (Dec. 2023) Targeting of the NOX1/ADAM17 Enzymatic Complex Regulates Soluble MCAM-Dependent Pro-Tumorigenic Activity in Colorectal Cancer. Biomed vol. 11(no. 12). 10.3390/BIOMEDICINES11123185 38137406 10.3390/biomedicines11123185 PMC10740863

[178] R Zhang et al (Aug. 2022) Inhibition of CD146 lessens uveal melanoma progression through reducing angiogenesis and vasculogenic mimicry. Cell Oncol vol. 45(no. 4):pp. 557–572. 10.1007/S13402-022-00682-9/FIGURES/6 10.1007/s13402-022-00682-9 PMC12978122 35716258

[179] L Zhang et al (Dec. 2018) CD146: a potential therapeutic target for systemic sclerosis. Protein Cell vol. 9(no. 12): pp. 1050–1054. 10.1007/S13238-018-0531-X 10.1007/s13238-018-0531-x PMC6251808 29671201

